# Compositing Benzothieno[3,2-*b*]Benzofuran Derivatives with Single-Walled Carbon Nanotubes for Enhanced Thermoelectric Performance

**DOI:** 10.3390/molecules28186519

**Published:** 2023-09-08

**Authors:** Yiyang Li, Liankun Ai, Qunyi Luo, Xin Wu, Baolin Li, Cun-Yue Guo

**Affiliations:** School of Chemical Sciences, University of Chinese Academy of Sciences, Beijing 100049, China; liyiyang211@mails.ucas.ac.cn (Y.L.); ailiankun19@mails.ucas.ac.cn (L.A.); luoqunyi20@mails.ucas.ac.cn (Q.L.); wuxin191@mails.ucas.ac.cn (X.W.)

**Keywords:** benzothieno[3,2-*b*]benzofuran, derivatives, single-walled carbon nanotubes, thermoelectric, composites

## Abstract

Although numerous thermoelectric (TE) composites of organic materials and single-walled carbon nanotubes (SWCNTs) have been developed in the past decade, most of the research has been related to polymers without much on organic small molecules (OSMs). In this work, benzothieno[3,2-*b*]benzofuran (BTBF) and its derivatives (BTBF-Br and BTBF-2Br) were synthesized and their TE composites with SWCNTs were prepared. It is found that the highest molecular orbital level and band gap (*E*_g_) of BTBF, BTBF-Br, and BTBF-2Br gradually decrease upon the introduction of electron-withdrawing Br group on BTBF. These changes significantly improve the Seebeck coefficient and power factor (PF) of OSM/SWCNT composites. An appropriate energy barrier between BTBF-2Br and SWCNTs promotes the energy filtering effect, which further contributes to the enhancement of composites’ thermoelectric properties. The composites of SWCNTs and BTBF-2Br with the smallest *E*_g_ (4.192 eV) afford the best thermoelectric performance with the room temperature power factor of 169.70 ± 3.46 μW m^−1^ K^−2^ in addition to good mechanical flexibility and thermal stability. This study provides a feasible strategy for the preparation of OSM/SWCNT composites with improved thermoelectric properties.

## 1. Introduction

In the face of the increasingly severe global energy crisis, reducing dependence on fossil energy and improving energy transformation efficiency have become the key [1]. Thermoelectric materials (TE) can convert heat into electricity through the migration of carriers inside a solid (the Seebeck effect), and have attracted great interest because they can be effectively used for waste heat recovery, and have a wide range of prospects for improving energy use efficiency and protecting the environment [2,3,4,5]. The performance of thermoelectric materials is mainly evaluated by a dimensionless figure of merit (*ZT* = *σS*^2^*T*/*κ*, where *σ*, *S*, *T*, and *κ* are electrical conductivity, the Seebeck coefficient (also called temperature difference electric potential), thermodynamic temperature, and thermal conductivity, respectively). For organic materials and organic/inorganic composites, power factor (PF = *σS*^2^) is adopted to assess thermoelectric properties of materials due to their inherent advantages of extremely low thermal conductivity in addition to difficulty in the measurement (thin films in particular). Ideal thermoelectric materials require excellent electrical conductivity, large Seebeck coefficient and low thermal conductivity at the same time [6,7,8,9,10]. Conventional inorganic thermoelectric materials such as Bi_2_Te_3_, Sb_2_Te_3_, and PbTe have excellent thermoelectric properties. However, inorganic materials have certain development bottlenecks and defects due to inherent disadvantages such as toxicity, high cost, inferior mechanical flexibility, and low abundance [11,12,13]. Organic thermoelectric materials have garnered much attention and become a hot spot in the field of thermoelectrics today because of their flexibility, low lattice thermal conductivity, and the ability to harvest energy at low temperatures. Simultaneously improving *S* and *σ* values is particularly challenging in one material, considering that these two parameters are interrelated [3]. To obtain materials with high thermoelectric properties, it has been proposed to compose interface-rich composites of conductive polymers (CPs) with inorganic fillers. The TE performance is improved by microstructural control and decoupling of parameters related to interfacial manipulation like in polymer/carbon nanofillers (e.g., CNT, graphene, GO) composites and polymer/inorganic TE nanoparticles composites elaborately reviewed by Nandihalli et al. [14,15]. SWCNTs have been widely used in thermoelectric composites due to their high electrical conductivity, low weight density, and flexibility. Current researches have focused on composites of CPs such as polyaniline (PANI), poly(3,4-ethylenedioxythiophene) (PEDOT), polypyrrole (PPy), and poly(3-hexylthiophene) (P3HT) with SWCNTs [16,17,18,19,20,21,22].

Compared to polymers, organic compounds with molecular weights below 900 Daltons, namely organic small molecules (OSMs), have advantages such as accurate structure, high purity, simple synthesis methods, and low cost. Therefore, OSMs have broad development space and huge development potential in the field of thermoelectricity [23]. The drawback of high thermal conductivity of SWCNTs can be successfully overcome by effectively wrapping the surface of SWCNTs with OSMs. The C-C bonds prevalent on the surface of SWCNTs can effectively interact with OSMs. Therefore, the thermoelectric properties of the composites prepared by physically blending OSMs with SWCNTs are significantly enhanced compared to those of pristine OSMs, which has become another feasible strategy for the preparation of high-performance thermoelectric composites [24,25,26,27,28]. Researchers have now synthesized a variety of desirable target molecules by chemical design and prepared OSM/SWCNT composites. For example, Chen et al. used thiazolo[3,4-*b*]pyrazine (TP) to synthesize TP dinitrogen oxide (TPNO) by in situ oxidation on the surface of SWCNTs, and the maximum PF of the composite is 29.4 ± 1.0 µW m^−1^ K^−2^ at room temperature. Zhou et al. prepared high-performance p-type thermoelectric composites containing metalloporphyrin molecules and SWCNTs, and SWCNT/ZnTPP composite film demonstrated the maximum PF of 247.2 μWm^−1^ K^−2^ at 340 K. Gao et al. prepared thermoelectric composites by mixing SWCNTs with liquid crystal mixture E7 at a mass ratio of 1:0.25, which reached a maximum PF of 428.59 ± 58.15 μW m^−1^ K^−2^ at room temperature [29,30,31]. 

The interaction between OSMs and SWCNTs and the mechanism affecting the thermoelectric properties of OSM/SWCNT composites have also attracted extensive interest from researchers. For example, Kim et al. investigated the effect of the highest occupied molecular orbital energy levels of OSMs on the thermoelectric properties of OSM/SWCNT composites. Yin et al. found that changes in the geometry and hybridization ratio of OSMs lead to significant changes in the morphology and grain boundaries of OSM/SWCNT composite films, which in turn affect the thermoelectric properties of the films [32,33,34,35]. In addition, theoretical study on OSM/SWCNT composites is helpful as in the case of using density-functional theory (DFT) on the investigation of electronic configuration of (*n*,*n*)-nanotubes by Jia’s group [36]. Some research progress has been made on OSM/SWCNT composites, but as it is, they still lag behind polymer/SWCNT composites and inorganic thermoelectric materials. In addition, the thermoelectric properties of most OSM/SWCNT composites are still lower than those of polymer/SWCNT composites and inorganic thermoelectric materials. How to improve the thermoelectric properties, in particular, the Seebeck coefficient of OSM/SWCNT composites, remains to be a hard nut [37,38,39]. Therefore, more efforts are still needed in the research and development of high-performance thermoelectric OSM/SWCNT composites, in addition to the exploration of the effect of OSMs’ structure and the interaction mechanism between OSMs and SWCNTs on thermoelectric properties.

Thiophene and furan-fused π-conjugated molecules have been viewed as very promising materials in organic electronics due to their good stability, high planarity, strong intermolecular π–π interaction, and excellent carrier transport properties [40]. In recent years, benzothieno[3,2-*b*]benzofuran (BTBF) has been found to be a promising candidate material [41,42,43,44,45]. For example, Wang et al. designed and synthesized 2,7-diphenylbenzo[4,5]thieno[3,2-*b*]benzothiophene (BTBF-DPh), which has high carrier mobility and strong emission properties [45]. However, to the best of our knowledge, BTBF and its derivatives have not been studied in thermoelectric applications, and BTBF might have great potential in the field of thermoelectricity due to its unique π-π conjugated structure and high mobility. Therefore, benzothieno[3,2-*b*]benzofuran, bromobenzothieno[3,2-*b*]benzofuran (BTBF-Br), and 2,7-dibromobenzo[4,5]thieno[3,2-*b*]benzofuran (BTBF-2Br) were synthesized in this work, and three types of composite films of OSMs and SWCNTs with different mass ratios were prepared, among which BTBF-2Br/SWCNT composites afforded the best thermoelectric performance. The 50 wt% BTBF-2Br/SWCNT composite film has a PF up to 169.70 ± 3.46 μW m^−1^ K^−2^, which is a good value among reported OSM/SWCNT thermoelectric composites (Table S1 in the Supplementary Materials). The effects of molecular structure, band gap, and energy level of BTBF and its derivatives on the thermoelectric properties of the composites were investigated. It is an inspiration for the preparation of subsequent OSM/SWCNT thermoelectric composites.

## 2. Results and Discussion

### 2.1. Structural Analyses of BTBF, BTBF-Br, and BTBF-2Br

**Figure 1:**
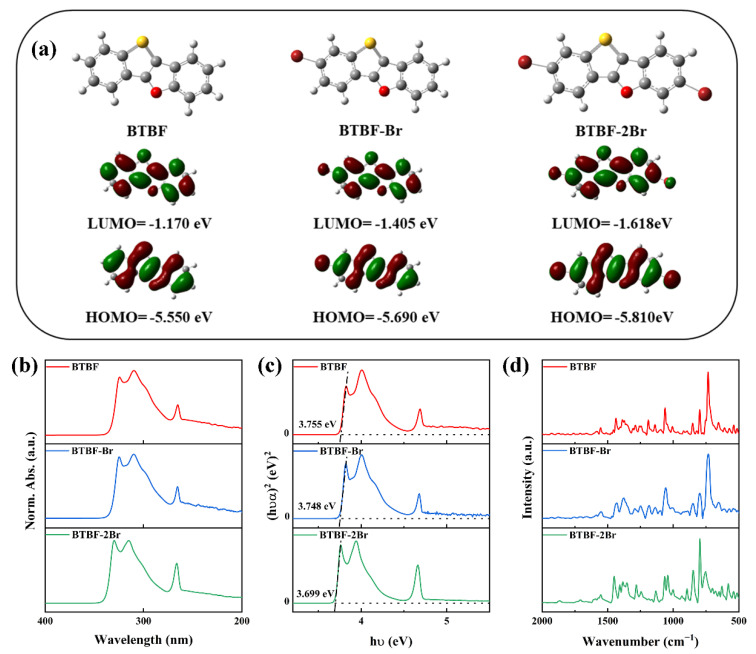
Molecular orbital energy levels (**a**) and UV-vis spectra (**b**) of BTBF, BTBF-Br, and BTBF-2Br. Tauc plot of (hνα)^2^ versus photon energy (hν) of BTBF, BTBF-Br, and BTBF-2Br based on UV-vis spectra (**c**). A linear fit was used to measure the bandgap by extrapolating to zero absorption. FT-IR spectra of BTBF, BTBF-Br, and BTBF-2Br (**d**).

The synthetic routes of BTBF, BTBF-Br, and BTBF-2Br, and HRMS spectra and ^1^H and ^13^C NMR spectra are included in the Supplementary Materials (SM). The HRMS spectra (Figures S13–S18), and ^1^H and ^13^C NMR spectra (Figures S1–S12) demonstrate the high purity of the prepared BTBF, BTBF-Br, and BTBF-2Br. The HOMO, LUMO energy levels and atomic orbitals of BTBF, BTBF-Br, and BTBF-2Br are predicted by DFT calculations (Figure 1a). The introduction of an electron-withdrawing Br group makes the HOMO and LUMO energy levels of as-synthesized BTBF, BTBF-Br, and BTBF-2Br decrease obviously. The introduction of the electron-withdrawing Br group directly affects the energy of the σ-orbitals and effectively reduces the electron density of the nuclei in the π-system. As a result, the electron cloud shielding of the p-orbitals constituting the π-system will be reduced, so that both the HOMO and LUMO energy levels tend to decrease [46]. The LUMO energy levels of BTBF-Br and BTBF-2Br drop to −1.405 and −1.618 eV, respectively. The HOMO energy levels drop less, reaching −5.690 eV for BTBF-Br and −5.810 eV for BTBF-2Br. In other words, since the HOMO energy levels of BTBF-Br and BTBF-2Br drop less than their LUMO energy levels, the band gap gradually decreases in the order of BTBF (4.380 eV) < BTBF-Br (4.285 eV) < BTBF-2Br (4.192 eV). As the HOMO energy level of the SWCNTs was known to be −5.060 eV, therefore, the HOMO energy level differences of SWCNT/BTBF, SWCNT/BTBF-Br, and SWCNT/BTBF-2Br were estimated to be 0.490, 0.630, and 0.75 eV, respectively.

UV-vis absorption spectra of three OSMs in dilute DMF solution were recorded and their optical band gaps were calculated. As shown in Figure 1b, all three OSMs in DMF solution showed strong absorption in the range of 250–350 nm, with typical structural vibrational absorption peaks and sharp absorption edges of π-conjugated molecules. The maximum absorption wavelengths (*λ*_max_, solu) of BTBF-Br and BTBF-2Br were 310 nm and 315 nm, respectively, which showed a red shift compared with the maximum absorption wavelength of BTBF (*λ*_max_ = 309 nm). The red shift for BTBF-Br and BTBF-2Br is attributed to the introduction of the conjugatively electron-donating Br group. The optical gaps of BTBF, BTBF-Br, and BTBF-2Br are 3.755, 3.748, and 3.699 eV, respectively (Figure 1c). These are basically consistent with the change trend of HOMO-LUMO gaps. In addition, FT-IR spectra likewise corroborated the effect of the introduction of the electron-withdrawing Br group on the electronic structure of the molecules. As indicated in Figure 1d, the maximum infrared absorption peak of BTBF occurs at 734 cm^−1^, and the maximum absorption peaks at 738 cm^−1^ for BTBF-Br and 799 cm^−1^ and ca. 752 cm^−1^ for BTBF-2Br are observed. The introduction of conjugatively electron-donating Br group, which leads to the contraction of the electron cloud, results in shorter bond lengths of C-S bond, higher bond energies, and increased vibrational frequencies of the chemical bonds. As a result, the infrared spectra of BTBF-Br and BTBF-2Br are blue-shifted compared to that of BTBF.

To evaluate the effect of doping of BTBF, BTBF-Br, and BTBF-2Br by SWCNTs, FT-IR measurements were performed on BTBF/SWCNT, BTBF-Br/SWCNT, and BTBF-2Br/SWCNT composites. As shown in Figures S19–S21, distinctive bands of BTBF, BTBF-Br, BTBF-2Br, and SWCNTs can be observed in the FT-IR spectra of the three OSM/SWCNT composite films. All three composites show double peaks at 2983 cm^−1^, 2987 cm^−1^ and single peak at 3676 cm^−1^, confirming the introduction of SWCNTs. The characteristic band of BTBF in BTBF/SWCNT composite is blue-shifted from 734 cm^−1^ to 783 cm^−1^, the characteristic band of BTBF-Br in BTBF-Br/SWCNT composite is blue-shifted from 738 cm^−1^ to 795 cm^−1^, and the characteristic band of BTBF-2Br in BTBF-2Br/SWCNT composite is blue-shifted from 752 cm^−1^ to 808 cm^−1^, indicating the formation of three composite films of OSMs and SWCNTs.

### 2.2. Morphological Observation and XPS Analyses of BTBF/SWCNT, BTBF-Br/SWCNT, and BTBF-2Br/SWCNT Composites

**Figure 2:**
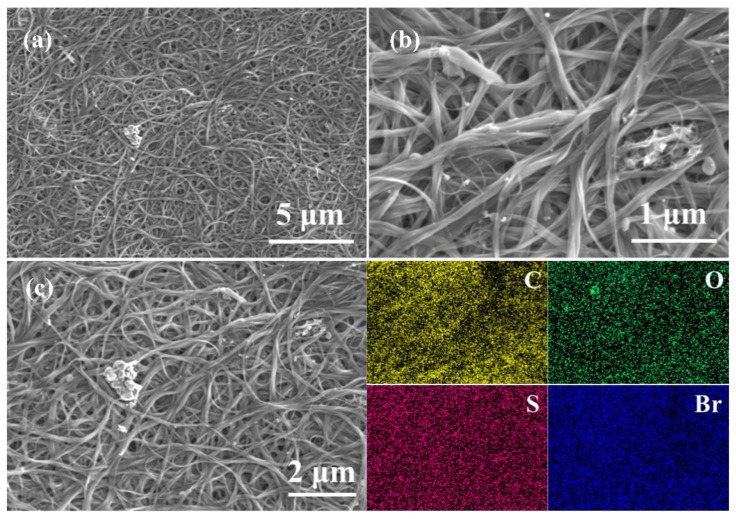
SEM images of 50 wt% BTBF-2Br/SWCNT composite film at 6000 (**a**), 30,000 magnification (**b**), and EDS mappings at 12,000 magnification (**c**).

The surface morphologies of three OSM/SWCNT composite films were observed on SEM, and EDS mappings were obtained under the condition of 15 kV to visually evaluate the distribution of OSMs in the composite films. In the SEM images of the three films (Figure 2, Figures S22 and S24), it can be seen that a large number of SWCNTs with a diameter of 1~2 nm are entangled with each other on the surface of the films, and there are OSMs clusters in some voids. EDS mappings (Figure 2c, Figures S23 and S25) of the three composite films show that S and O elements are uniformly distributed in the film, and Br element is also uniformly distributed in the BTBF-Br/SWCNT and BTBF-2Br/SWCNT composite films. These results indicate uniform dispersion of OSMs and their combination with SWCNTs in the formation of composite films.

### 2.3. Thermoelectric Properties of BTBF/SWCNT, BTBF-Br/SWCNT, and BTBF-2Br/SWCNT Composites

**Figure 3:**
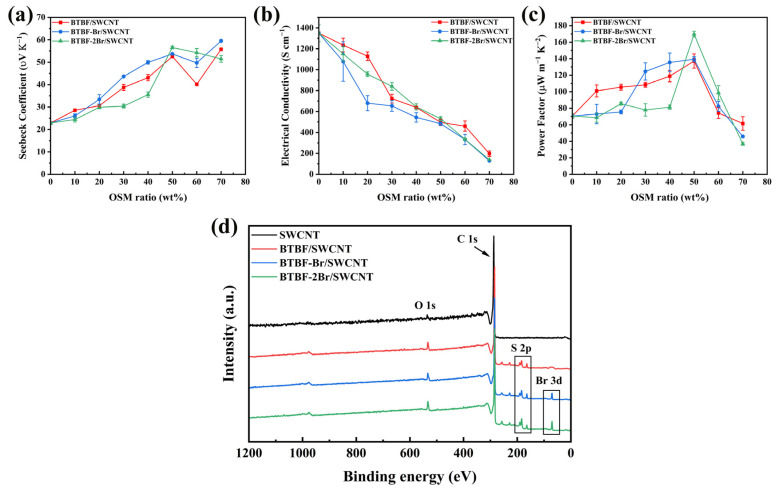
(**a**) Seebeck coefficients, (**b**) electrical conductivities, and (**c**) PF of BTBF/SWCNT, BTBF-Br/SWCNT, and BTBF-2Br/SWCNT with different concentrations of OSMs. (**d**) XPS spectra of pure SWCNT, 50 wt% BTBF/SWCNT, BTBF-Br/SWCNT, and BTBF-2Br/SWCNT.

Composite films were prepared by evaporating the solvent after compositing OSMs with SWCNTs. The content of OSMs in the composites are set at 10~70 wt% with an interval of 10 wt%. As shown in Figure 3a–c, the thermoelectric properties of the three OSM/SWCNT composite films show a similar variation trend with the increase of the OSMs content in the composite films, and the highest PF is obtained at 50 wt% OSMs in the composites.

XPS spectra of the composites and SWCNTs are displayed in Figure 3d. Unlike SWCNTs, all three OSM/SWCNT composite films have distinct peaks at 164 eV which are assigned to S 2p of the three OSMs. And the BTBF-Br/SWCNT and BTBF-2Br/SWCNT composite films also have peaks at 70 eV, which are assigned to Br 3d of BTBF-Br and BTBF-2Br. This indicates the existence of constituent elements and the interaction among them in composite films.

The Seebeck coefficient increases with rising OSMs content in OSM/SWCNT composite films in the range of 10 to 50 wt%, while the conductivity decreases gradually. However, further increase of the OSMs content to 60 and 70 wt% results in significant decrease in thermoelectric properties of the OSM/SWCNT composite films. The electrical conductivity of BTBF/SWCNT composite film decreases from 1235.24 ± 65.56 S cm^−1^ to 197.50 ± 25.46 S cm^−1^, the conductivity of BTBF-Br/SWCNT composite film decreased from 1078.00 ± 188.82 S cm^−1^ to 129.25 ± 4.62 S cm^−1^, and the conductivity of BTBF-2Br/SWCNT composite film decreased from 1150.69 ± 67.96 S cm^−1^ to 138.44 ± 2.22 S cm^−1^. The Seebeck coefficient of BTBF/SWCNT and BTBF-Br/SWCNT composite films has two peak values with 52.57 ± 0.32 μV K^−1^ at 50 wt% BTBF and 55.76 ± 0.39 μV K^−1^ at 70 wt% BTBF and 53.67 ± 0.24 μV K^−1^ at 50 wt% BTBF-Br and 59.51 ± 0.43 μV K^−1^ at 70 wt% BTBF-Br. In contrast, the Seebeck coefficient of BTBF-2Br/SWCNT composite film reaches its peak of 56.55 ± 0.58 μV K^−1^ when the content of BTBF-2Br is 50 wt%. Although the increase of OSMs content leads to the decline in the conductivity of OSM/SWCNT composite films, the PF value of OSM/SWCNT composite films determined by *S*^2^*σ* rise remarkably with the increase of the Seebeck coefficient. As a result, the room temperature PF of 50 wt% BTBF/SWCNT composite film is 137.12 ± 8.57 μW m^−1^ K^−2^ and that of 50 wt% BTBF-Br/SWCNT composite film is 139.26 ± 3.00 μW m^−1^ K^−2^. The 50 wt% BTBF-2Br/SWCNT composite film has a PF of 169.70 ± 3.46 μW m^−1^ K^−2^, which is the highest among three composite films. This indicates that changing the mass ratio of OSMs in OSM/SWCNT composites can significantly affect the thermoelectric properties of the composite films.

### 2.4. Raman and Ultraviolet Photoelectron Spectral Investigation of BTBF/SWCNT, BTBF-Br/SWCNT, and BTBF-2Br/SWCNT Composites

**Figure 4:**
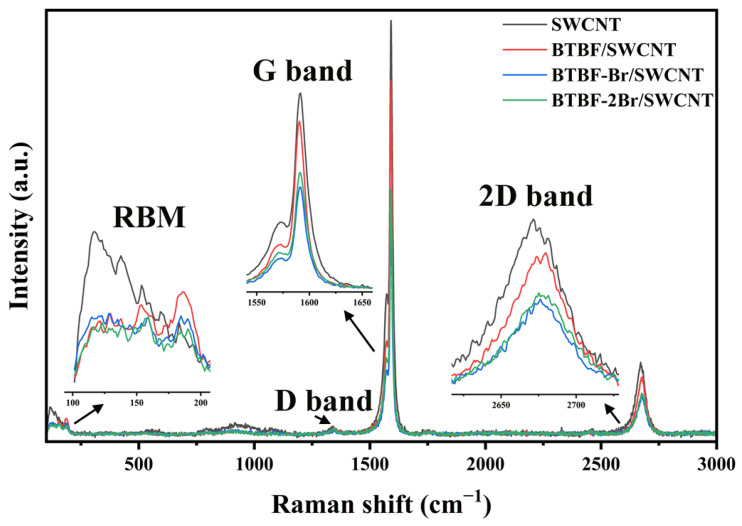
Raman spectra of pristine SWCNTs, 50 wt% BTBF/SWCNT, BTBF-Br/SWCNT, and BTBF-2Br/SWCNT composites.

In order to further probe the interaction between OSMs and SWCNTs in OSM/SWCNT composites and the structural integrity of SWCNTs, Raman spectra are recorded for SWCNTs, 50 wt% BTBF/SWCNT, 50 wt% BTBF-Br/SWCNT, and 50 wt% BTBF-2Br/SWCNT composites. As shown in Figure 4, the radial breathing mode (RBM), D, G, and 2D bands can be clearly observed in the Raman spectra of SWCNTs. The RBM band at 100~200 cm^−1^ indicates synchronous radial vibration of carbon atoms. The RBM band intensity of the three 50 wt% OSM/SWCNT composite films is lower than that of SWCNTs, which could be attributed to the decrease of absolute content of SWCNTs in composites. The characteristic G band is located at 1572 cm^−1^ (G^−^ mode) and 1590 cm^−1^ (G^+^ mode), which are mainly used to assess the orderliness of the SWCNTs arrangement. In the Raman spectra of the three 50 wt% OSM/SWCNT composite films, the intensities of the characteristic G band show a decrease, but the band shapes remain unchanged with regard to that of the SWCNT composite films. The addition of OSMs does not affect the structural orderliness of SWCNTs. And due to the π-π conjugation interactions between the three OSMs and SWCNTs, the characteristic G bands of all three 50 wt% OSM/SWCNT composite films display a small blue shift. The G band of the 50 wt% BTBF/SWCNT film appears at 1591 cm^−1^, while the G bands of the 50 wt% BTBF-Br/SWCNT and BTBF-2Br/SWCNT composite films appear at 1593 cm^−1^. The characteristic D and 2D bands of the SWCNTs are located at 1340 and 2671 cm^−1^, respectively, while the two bands of the three 50 wt% OSM/SWCNT composites also show a slight blue shift as well as a decrease in intensity. The 2D band of the 50 wt% BTBF/SWCNT composite film appears at 2673 cm^−1^, whereas the 2D bands of the 50 wt% BTBF-Br/SWCNT and the BTBF-2Br/SWCNT composite films appears at 2676 cm^−1^. The G/D ratios for the three 50 wt% OSM/SWCNT composite films are essentially similar to that of pristine SWCNTs, suggesting that the addition of OSMs does not cause structural defects in the SWCNTs. On the other hand, all three 50 wt% OSM/SWCNT composite films show a blue shift as well as decrease in intensity in the RBM, G, and 2D bands, but the change in the spectra of the 50 wt% BTBF-Br/SWCNT and BTBF-2Br/SWCNT composite films is much bigger than that of the 50 wt% BTBF/SWCNT composite films. This is due to the strong hydrophobicity of SWCNTs, and the introduction of the hydrophobic Br group promotes the dispersion of BTBF-Br and BTBF-2Br in SWCNTs and enhances π-π conjugation interactions between Br-substituted BTBFs and SWCNTs according to the principle of similar phase solubility [32].

In addition, Raman spectra of BTBF/SWCNT, BTBF-Br/SWCNT, and BTBF-2Br/SWCNT composite films at 10 wt% and 30 wt% OSMs were also analyzed. As shown in Figure S26, the G/D ratios related to SWCNTs in the three OSM/SWCNT composite films do not change significantly with increasing content of OSMs. This suggests that OSMs as organic components have negligible influence on the structural integrity of SWCNTs during the formation of composites with SWCNTs.

**Figure 5:**
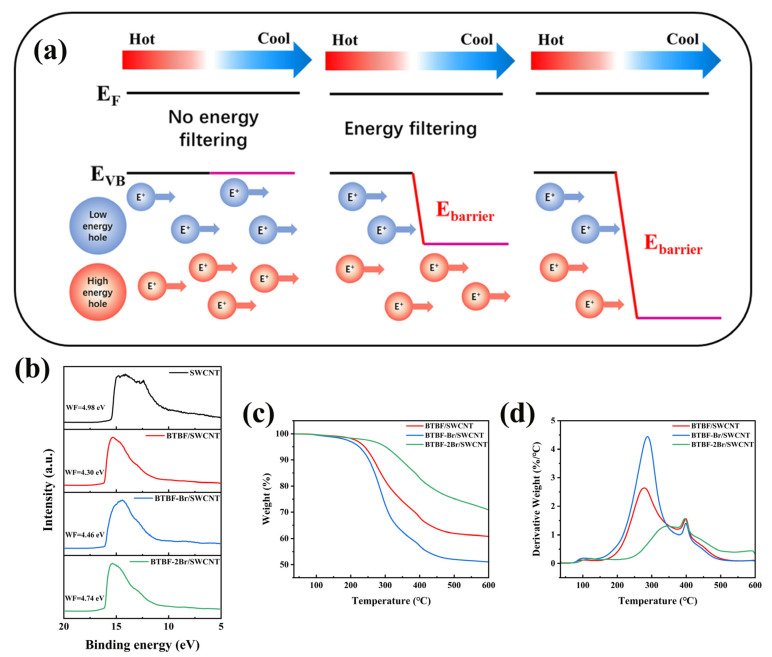
Schematic illustration of the energy-filtering effect (**a**), UPS spectra (**b**), TGA (**c**), and DTG curves (**d**) of 50 wt% BTBF/SWCNT, BTBF-Br/SWCNT, and BTBF-2Br/SWCNT composites.

The work functions (*W*_F_) of SWCNTs and three 50 wt% OSM/SWCNT composite films are calculated by utilizing UPS spectra (Figure 5b) with which the effect of the introduction of three OSMs on the electronic structure of the composite films is evaluated. As shown in Figure 5b, the *W*_F_ of the three OSM/SWCNT composite films decreases compared to the value of 4.98 eV for SWCNTs. The *W*_F_ of BTBF/SWCNT, BTBF-Br/SWCNT, and BTBF-2Br/SWCNT composite films are 4.30, 4.46, and 4.74 eV, respectively. The decrease in the *W*_F_ of the three OSM/SWCNT composite films proves that the Fermi energy level (*E*_F_) in the band gap of the composites shifts upward, leading to a decrease in the hole carrier density, which contributes to the improvement of the Seebeck coefficient. The difference between the *W*_F_ of the three OSM/SWCNT films and the SWCNTs (Δ*W*_F_ for BTBF/SWCNT = 0.68 eV, Δ*W*_F_ for BTBF-Br/SWCNT = 0.52 eV, and Δ*W*_F_ for BTBF-2Br/SWCNT = 0.24 eV) can reflect the energy barriers of the three OSMs and SWCNTs. Although BTBF-2Br/SWCNT composite has the highest *W*_F_, it is closest to that of SWCNTs, which indicates the lowest energy barrier between BTBF-2Br and SWCNTs. According to the rule of energy filtering, the aggregation of low-energy carriers at the cold end is blocked and the average energy of holes aggregated at the cold end is increased due to the existence of energy barriers between OSMs and SWCNTs [47,48]. Meanwhile, since the Seebeck coefficient is proportional to the average energy of the holes aggregated at the cold end, filtering out the low-energy holes can significantly increase the Seebeck coefficient. The energy barrier between BTBF-2Br and SWCNTs is minimized, which has less effect on the concentration of carriers, thus improving the overall thermoelectric properties of the composite to the largest extent.

The carrier concentration and mobility of three 50 wt% OSM/SWCNT composite films are measured by Hall effect tests. Due to the energy filtering effect, energy barriers between the three OSMs and SWCNTs prevent the migration of low-energy holes from the hot end to the cold end and improve the Seebeck coefficients of the three OSM/SWCNT composite films. Therefore, an excessively high energy barrier for BTBF-SWCNTs and BTBF-Br-SWCNTs similarly leads to a decrease in carrier concentration. An appropriate and relatively low energy barrier between BTBF-2Br and SWCNTs can allow high-energy hole movement and prevent low-energy hole movement, which leads to a high carrier concentration in BTBF-2Br/SWCNT composite films [48,49]. An increase in carrier concentration leads to a decrease in carrier mobility due to the effect of scattering between carriers. The result agrees well with the *W*_F_ data.

As shown in Table 1, The BTBF-2Br/SWCNT composite film has the highest carrier concentration (2.94 × 10^20^ cm^−3^), twice that of the BTBF/SWCNT composite film and three times that of the BTBF-Br/SWCNT composite film. The highest electrical conductivity (530.86 S cm^−1^) originated from the biggest carrier concentration of the 50 wt% BTBF-2Br/SWCNT composite, together with its highest Seebeck coefficient (59.51 ± 0.43 μV K^−1^), and moderate carrier mobility gives rise to the best thermoelectric performance (PF = 169.70 ± 3.46 μW m^−1^ K^−2^).

### 2.5. Thermal Stability and Flexibility of BTBF/SWCNT, BTBF-Br/SWCNT, and BTBF-2Br/SWCNT Composites

To investigate the thermal stability of OSM/SWCNT composite films at high temperatures, the 50 wt% OSM/SWCNT composite films were subjected to thermogravimetric tests in the range of room temperature to 600 °C with a heating rate of 10 °C min^−1^. As shown in Figure 5c,d, the *T*_d_ (temperature at 5% weight loss) of BTBF-2Br/SWCNT is 300 °C, which is higher than those of BTBF/SWCNT (240 °C) and BTBF-Br/SWCNT composites (224 °C). In addition, the *T*_max_ (temperature with maximum mass loss rate) of BTBF-2Br/SWCNT composite film is 396 °C, well exceeding those of BTBF/SWCNT (278 °C) and BTBF-Br/SWCNT composite films (287 °C). At 600 °C, the mass remaining in the samples is 60% for BTBF/SWCNT, 50% for BTBF-Br/SWCNT, and 70% for BTBF-2Br/SWCNT composite films. This indicates that all three OSM/SWCNT films have good thermal stability, among which the BTBF-2Br/SWCNT composite ranks the best. Such thermal stability will benefit potential application of these thermoelectric composites in harvesting low-grade thermal energy.

In practical scenarios, numerous heat sources have uneven surfaces. Therefore, flexible thermoelectric materials are expected to create conformal contact between heat sources and the materials. As for 50 wt% BTBF-2Br/SWCNT composite films, 25, 50, and 100 bending cycles were performed at a bending radius of 10 mm to test their mechanical flexibility. As shown in Table 2, the Seebeck coefficient of the sample is almost unchanged after 100 bending cycles, but its electrical conductivity decreases by 24%. As a result, 70% of its original PF is maintained after 100 bending cycles, indicating relatively good flexibility. Although the flexibility of such composite film is still inferior to most of that of polymer/SWCNT composite films, continuing optimization in the compositing process will further enlarge this advantage in addition to other virtues of OSM/SWCNT thermoelectric composites.

## 3. Experimental Section

### 3.1. Chemicals and Materials

BTBF, BTBF-Br, and BTBF-2Br used in this study for the preparation of OSM/SWCNT composite films were synthesized through intramolecular dehydrogenation C-H/O-H coupling by using chemicals as elaborated in the Supplementary Materials and our previous work [42,43,50]. Commercially available SWCNTs with large specific surface area (95% purity, XFNANO Nanomaterials Technology Co., Ltd., Nanjing, China) were used for the preparation of OSM/SWCNT composite films. The solvent used was commercially available biotech-grade *N*,*N*’-dimethylformamide (DMF, 99% purity, Macklin Reagent, Shanghai, China).

### 3.2. Preparation of Composite Films

Six mg of SWCNTs were put into 25 mL of DMF with BTBF, BTBF-Br and BTBF-2Br, respectively, and the OSM/SWCNT composite films with different mass ratios were obtained by varying the mass of OSMs added. After that, the mixed suspension was sonicated for 90 min using a JY96-11N (SCIENTZ Ltd., Ningbo, China) at 65 W, and the sonicated OSM/SWCNT dispersion was poured into the Teflon molds cleaned by anhydrous ethanol and pure water, and finally put into an electric thermostatic blast dryer to prepare the films by drying the solvent at 60 °C for 24 h.

### 3.3. Analyses and Characterization

Proton and carbon nuclear magnetic resonance (^1^H NMR and ^13^C NMR) spectra were tested using a JNM-ECZ400S (JEOL Ltd., Tokyo, Japan) with CDCl_3_ as solvent. The high-resolution mass spectra (HRMS) were tested by a Q Exactive Focus (Thermo Fisher Scientific Inc., Waltham, MA, USA). Ultraviolet-visible (UV-vis) absorption spectra were measured by a UV-2600 (Shimadzu Ltd., Kyoto, Japan). The lowest unoccupied molecular orbital (LUMO) energy levels were estimated from empirical highest occupied molecular orbital (HOMO) energy levels and optical band gaps. DFT calculations were performed using Gaussian 09 at the B3LYP/6-31G* level.

Fourier-infrared (FT-IR) spectra were tested using a VERTE-70 (Bruker Ltd., Ettlingen, Germany). Raman spectra were tested using a 532 nm-in Via (Renishaw Ltd., Sheffield, UK). X-ray photoelectron and ultraviolet photoelectron (XPS and UPS) spectra were tested using Axis Supra (Shimadzu Ltd., Japan). Scanning electron microscope and energy dispersive spectra (SEM and EDS) maps were tested using a SU8010 (HITACHI Ltd., Tokyo, Japan). The thermal stability of the films was tested using a STA200 (HITACHI Ltd., Japan). Hall effect measurements were performed using a HET-3RT (JouleYacht Ltd., Wuhan, China). The thermoelectric properties of the films were tested at ambient and elevated temperatures using the MRS-3RT (Joule Yacht Ltd., China).

## 4. Conclusions

In summary, we synthesize zothieno[3,2-*b*]benzofuran (BTBF) and its two derivatives (BTBF-Br and BTBF-2Br), based on which BTBF/SWCNT, BTBF-Br/SWCNT, and BTBF-2Br/SWCNT composite films with enhanced thermoelectric properties are prepared. Structural analyses find that the introduction of an electron-withdrawing Br group significantly changed the HOMO and LUMO energy levels of BTBF. On one hand, the hydrophobic Br moiety can increase the π-π conjugation interaction between OSMs and SWCNTs and promote the dispersion of OSMs in OSM/SWCNT composite films. On the other hand, the energy filtering effect due to the energy barrier between BTBF-2Br and SWCNTs greatly lifts the Seebeck coefficient and corresponding PF of the OSM/SWCNT composites. The 50 wt% BTBF-2Br/SWCNT composite affords the best thermoelectric properties with electrical conductivity of 530.86 ± 18.72 S cm^−1^, a Seebeck coefficient of 56.55 ± 0.58 μV K^−1^, and a PF value of 169.70 ± 3.46 μW m^−1^ K^−2^ at room temperature. In addition, all three OSM/SWCNT composites are thermally stable with the best properties for the 50 wt% BTBF-2Br/SWCNT composite. Moreover, the 50 wt% BTBF-2Br/SWCNT composite film is flexible and 70% of its initial PF can be maintained after 100 bending cycles, manifesting potentials in harvesting thermal energy with irregular heat source surfaces. Our findings expand the scope of OSMs which are promising as potential components in the formation of thermoelectric materials. Such flexible composite films are suitable for fabricating wearable self-powered electronics in the case of harvesting heat from sources with irregular surfaces. Although BTBF-2Br/SWCNT composite affords good thermoelectric performance, there still leaves large room for improvement with respect to its mechanical flexibility, optimization of processing parameters in preparing OSM/SWCNT composite films, and theoretical study on the composites. It is expected that continuing research in OSM/SWCNT composites will gradually shorten their gap with polymer/SWCNT composites and there is one day OSMs can seal a place and make exclusive contribution on par with polymer/SWCNT composites to the development of flexible thermoelectric materials.

## Figures and Tables

**Table 1:** Carrier concentration (*n*), electrical conductivity (*σ*), and carrier mobility (*μ*) of the 50 wt% BTBF/SWCNT, BTBF-Br/SWCNT, and BTBF-2Br/SWCNT composites.

Film Name	*n* (cm^−3^)	*μ* (cm^2^ V^−1^ s^−1^)	*σ* (S cm^−1^)
BTBF/SWCNT	1.48 × 10^20^	15.8	496.05
BTBF-Br/SWCNT	9.43 × 10^19^	17.0	483.47
BTBF-2Br/SWCNT	2.94 × 10^20^	11.0	530.86

**Table 2:** Thermoelectric properties of 50 wt% BTBF-2Br/SWCNT composite films after multiple bending.

Bending Cycle	*S* (μV K^−1^)	*σ* (S cm^−1^)	PF (μW m^−1^ K^−2^)
0	59.51	530.86	169.70
25	56.68	495.50	159.18
50	55.13	468.45	142.37
100	54.07	399.01	116.65

## Data Availability

Not applicable.

## Supplementary Materials

The following supporting information can be downloaded at: https://www.mdpi.com/article/10.3390/molecules28186519/s1, Figure S1: ^1^H NMR spectrum of 1. Figure S2: ^13^C NMR spectrum of 1. Figure S3: ^1^H NMR spectrum of 2 (BTBF). Figure S4: ^13^C NMR spectrum of 2 (BTBF). Figure S5: ^1^H NMR spectrum of 3 (BTBF-Br). Figure S6: ^13^C NMR spectrum of 3 (BTBF-Br). Figure S7: ^1^H NMR spectrum of 4. Figure S8: ^13^C NMR spectrum of 4. Figure S9: ^1^H NMR spectrum of 5. Figure S10: ^13^C NMR spectrum of 5. Figure S11: ^1^H NMR spectrum of 6 (BTBF-2Br). Figure S12: ^13^C NMR spectrum of 6 (BTBF-2Br). Figure S13: HRMS spectrum of 1. Figure S14: HRMS spectrum of 2 (BTBF). Figure S15: HRMS spectrum of 3 (BTBF-Br). Figure S16: HRMS spectrum of 4. Figure S17: HRMS spectrum of 5. Figure S18: HRMS spectrum of 6 (BTBF-2Br). Figure S19: FT-IR spectra of BTBF/SWCNT composite film. Figure S20: FT-IR spectra of BTBF-Br/SWCNT composite film. Figure S21: FT-IR spectra of BTBF-2Br/SWCNT composite film. Figure S22: SEM images of 50 wt% BTBF/SWCNT composite film at 6 k (a), 12 k (b), and 30 k (c) magnification. Figure S23: EDS mappings of 50 wt% BTBF/SWCNT at 12 k magnification. Figure S24: SEM images of 50 wt% BTBF-Br/SWCNT composite film at 6000 (a), 12,000 (b), and 30,000 (c) magnification. Figure S25: EDS mappings of 50 wt% BTBF-Br/SWCNT at 12 k magnification. Figure S26: Raman spectra of pure SWCNT, BTBF/SWCNT (a), BTBF-Br/SWCNT (b), and BTBF-2Br/SWCNT (c). Table S1. Comparison of thermoelectric properties of representative OSM/SWCNT composites at room temperature. Ref. [51] is cited in the Supplementary Materials.

## Sample Availability

Samples of the compounds are not available from the authors.

## References

[1] Chow J., Kopp R.J., Portney P.R. (2003). Energy resources and global development. Science.

[2] Brantut J.P., Grenier C., Meineke J., Stadler D., Krinner S., Kollath C., Esslinger T., Georges A. (2013). A thermoelectric heat engine with ultracold atoms. Science.

[3] Russ B., Glaudell A., Urban J.J., Chabinyc M.L., Segalman R.A. (2016). Organic thermoelectric materials for energy harvesting and temperature control. Nat. Rev. Mater..

[4] Du Y., Shen S.Z., Cai K., Casey P.S. (2012). Research progress on polymer–inorganic thermoelectric nanocomposite materials. Prog. Polym. Sci..

[5] Vining C.B. (2009). An inconvenient truth about thermoelectrics. Nat. Mater..

[6] Mo J.-H., Kim J.-Y., Kang Y.H., Cho S.Y., Jang K.-S. (2018). Carbon Nanotube/Cellulose Acetate Thermoelectric Papers. ACS Sustain. Chem. Eng..

[7] Liu J., Qiu L., Portale G., Koopmans M., Ten Brink G., Hummelen J.C., Koster L.J.A. (2017). N-type organic thermoelectrics: Improved power factor by tailoring host-dopant miscibility. Adv. Mater..

[8] Jiang B., Yu Y., Cui J., Liu X., Xie L., Liao J., Zhang Q., Huang Y., Ning S., Jia B. (2021). High-entropy-stabilized chalcogenides with high thermoelectric performance. Science.

[9] Biswas K., He J., Zhang Q., Wang G., Uher C., Dravid V.P., Kanatzidis M.G. (2011). Strained endotaxial nanostructures with high thermoelectric figure of merit. Nat. Chem..

[10] He J., Tritt T.M. (2017). Advances in thermoelectric materials research: Looking back and moving forward. Science.

[11] Gayner C., Kar K.K. (2016). Recent advances in thermoelectric materials. Prog. Mater. Sci..

[12] Venkatasubramanian R., Siivola E., Colpitts T., O’quinn B. (2001). Thin-film thermoelectric devices with high room-temperature figures of merit. Nature.

[13] Chang C., Wu M., He D., Pei Y., Wu C.F., Wu X., Yu H., Zhu F., Wang K., Chen Y. (2018). 3D charge and 2D phonon transports leading to high out-of-plane ZT in n-type SnSe crystals. Science.

[14] Nandihalli N. (2023). Imprints of interfaces in thermoelectric materials. Crit. Rev. Solid State.

[15] Nandihalli N., Liu C.J., Mori T. (2020). Polymer based thermoelectric nanocomposite materials and devices: Fabrication and characteristics. Nano Energy.

[16] Yin S., Lu W., Wu X., Luo Q., Wang E., Guo C.Y. (2021). Enhancing thermoelectric performance of polyaniline/single-walled carbon nanotube composites via dimethyl sulfoxide-mediated electropolymerization. ACS Appl. Mater. Interfaces.

[17] Wu X., Yin S., Guo C.-Y. (2022). Self-healable and robust PE/PEDOT/SWCNT thermoelectric composites. ACS Appl. Mater. Interfaces.

[18] Yin S., Lu W., Wu R., Fan W., Guo C.Y., Chen G. (2020). Poly(3,4-ethylenedioxythiophene)/Te/single-walled carbon nanotube composites with high thermoelectric performance promoted by electropolymerization. ACS Appl. Mater. Interfaces.

[19] Fan W., Guo C.-Y., Chen G. (2018). Flexible films of poly(3,4-ethylenedioxythiophene)/carbon nanotube thermoelectric composites prepared by dynamic 3-phase interfacial electropolymerization and subsequent physical mixing. J. Mater. Chem. A.

[20] Fan W., Liang L., Zhang B., Guo C.-Y., Chen G. (2019). PEDOT thermoelectric composites with excellent power factors prepared by 3-phase interfacial electropolymerization and carbon nanotube chemical doping. J. Mater. Chem. A.

[21] Lu W., Yin S., Wu X., Luo Q., Wang E., Cui L., Guo C.-Y. (2021). Aniline–pyrrole copolymers formed on single-walled carbon nanotubes with enhanced thermoelectric performance. J. Mater. Chem. C.

[22] Lee W., Hong C.T., Kwon O.H., Yoo Y., Kang Y.H., Lee J.Y., Cho S.Y., Jang K.S. (2015). Enhanced thermoelectric performance of bar-coated SWCNT/P3HT thin films. ACS Appl. Mater. Interfaces.

[23] Zhang Q., Sun Y., Xu W., Zhu D. (2014). Organic thermoelectric materials: Emerging green energy materials converting heat to electricity directly and efficiently. Adv. Mater..

[24] Bin J.K., Cho N.S., Hong J.I. (2012). New host material for high-performance blue phosphorescent organic electroluminescent devices. Adv. Mater..

[25] Lee W., Lim J., Lee J.-K., Hong J.-I. (2014). Oligothiophene-modified silver/silica core–shell nanoparticles for inhibiting open-circuit voltage drop and aggregation in polymer solar cells. J. Mater. Chem. A.

[26] Li X., Yu Z., Zhou H., Yang F., Zhong F., Mao X., Li B., Xin H., Gao C., Wang L. (2021). Promoting the thermoelectric performance of single-walled carbon nanotubes by inserting discotic liquid-crystal molecules. ACS Sustain. Chem. Eng..

[27] Jeon Y., Jang J.G., Kim S.H., Hong J.-I. (2021). Twisted small organic molecules for high thermoelectric performance of single-walled carbon nanotubes/small organic molecule hybrids through mild charge transfer interactions. J. Mater. Chem. C.

[28] Wang D., Li J., Yang K., Wang Y., Jeong S.Y., Chen Z., Liao Q., Li B., Woo H.Y., Deng X. (2023). Terminal cyano-functionalized fused bithiophene imide dimer-based n-type small molecular semiconductors: Synthesis, structure-property correlations, and thermoelectric performances. ACS Appl. Mater. Interfaces.

[29] Gao C., Chen G. (2018). In situ oxidation synthesis of p-type composite with narrow-bandgap small organic molecule coating on single-walled carbon nanotube: Flexible film and thermoelectric performance. Small.

[30] Zhou Y., Liu Y., Zhou X., Gao Y., Gao C., Wang L. (2019). High performance p-type organic thermoelectric materials based on metalloporphyrin/single-walled carbon nanotube composite films. J. Power Sources.

[31] Nie X., Li X., Huang Y., Wu J., Yang F., Zhong F., Hong X., Gao C., Wang L. (2021). High performance of p-type and n-type thermoelectric materials based on liquid crystal mixture and single-walled carbon nanotube composites. Compos. Commun..

[32] Zhou Y., Yin X., Liu Y., Zhou X., Wan T., Wang S., Gao C., Wang L. (2019). Significantly enhanced power factors of p-type carbon nanotube-based composite films by tailoring the peripheral substituents in porphyrin. ACS Sustain. Chem. Eng..

[33] Kim T.-H., Jang J.G., Hong J.-I. (2020). Enhanced thermoelectric performance of SWNT/organic small molecule (OSM) hybrid materials by tuning of the energy level of OSMs. J. Mater. Chem. C.

[34] Yin X., Peng Y., Luo J., Zhou X., Gao C., Wang L., Yang C. (2018). Tailoring the framework of organic small molecule semiconductors towards high-performance thermoelectric composites via conglutinated carbon nanotube webs. J. Mater. Chem. A.

[35] Kumari A., Ghosh A., Mehta B.R., Sinha A. (2023). Synergetic enhancement of Seebeck coefficients and electrical conductivity in flexible liquid crystal composites. ACS Sustain. Chem. Eng..

[36] Luo H.C., Li F.Y., Zhang Y.N., Zhang H.X., Eglitis R.I., Jia R. (2023). Theoretical study on (*n*,*n*)-nanotubes rolled-up from B/N substituted Me-graphene. Crystals.

[37] Kim T.H., Hong J.I. (2022). Energy level modulation of small molecules enhances thermoelectric performances of carbon nanotube-based organic hybrid materials. ACS Appl. Mater. Interfaces.

[38] Wei L., Huang H., Gao C., Liu D., Wang L. (2021). Novel butterfly-shaped organic semiconductor and single-walled carbon nanotube composites for high performance thermoelectric generators. Mater. Horiz..

[39] Wang Y., Chen Z., Huang H., Wang D., Liu D., Wang L. (2020). Organic radical compound and carbon nanotube composites with enhanced electrical conductivity towards high-performance p-type and n-type thermoelectric materials. J. Mater. Chem. A.

[40] Takimiya K., Osaka I., Mori T., Nakano M. (2014). Organic semiconductors based on [1]benzothieno [3,2-*b*][1]benzothiophene substructure. Acc. Chem. Res..

[41] Tsuji H., Nakamura E. (2017). Design and functions of semiconducting fused polycyclic furans for optoelectronic applications. Acc. Chem. Res..

[42] Chen D., Yuan D., Zhang C., Wu H., Zhang J., Li B., Zhu X. (2017). Ullmann-type intramolecular C–O reaction toward thieno[3,2-*b*]furan derivatives with up to six fused rings. J. Org. Chem..

[43] Ma W., Huang J., Li C., Jiang Y., Li B., Qi T., Zhu X. (2019). One-pot synthesis and property study on thieno[3,2-*b*]furan compounds. RSC Adv..

[44] Krishnan R.A., Babu S.A., Nitha P.R., Krishnan J., John J. (2021). Synthesis of benzothienobenzofurans via annulation of electrophilic benzothiophenes with phenols. Org. Lett..

[45] Wang J., He Y., Guo S., Ali M.U., Zhao C., Zhu Y., Wang T., Wang Y., Miao J., Wei G. (2021). Multifunctional benzo[4,5]thieno[3,2-*b*]benzofuran derivative with high mobility and luminescent properties. ACS Appl. Mater. Interfaces.

[46] Anthony J.E., Facchetti A., Heeney M., Marder S.R., Zhan X. (2010). n-Type organic semiconductors in organic electronics. Adv. Mater..

[47] Zhang L., Jin J., Huang S., Tan B., Luo J., Wang D., Liu D., Wang L. (2021). Cross-conjugated spiro molecules and single-walled carbon nanotubes composite for high-performance organic thermoelectric materials and generators. Chem. Eng. J..

[48] Guan X., Ouyang J. (2021). Enhancement of the Seebeck coefficient of organic thermoelectric materials via energy filtering of charge carriers. CCS Chem..

[49] Lu N., Li L., Liu M. (2016). A review of carrier thermoelectric-transport theory in organic semiconductors. Phys. Chem. Chem. Phys..

[50] Ai L., Ajibola I.Y., Li B. (2021). Copper-mediated construction of benzothieno[3,2-*b*]benzofurans by intramolecular dehydrogenative C-O coupling reaction. RSC Adv..

[51] Ai L., Xie X., Li B., Wang Y. (2023). Pure blue emitters based on benzo[4,5]thieno-S,S-dioxide-[3,2-*b*]benzofuran with high thermal stability. Dye. Pigment..

