# Supplementary material for: Compositing Benzothieno[3,2-b]Benzofuran Derivatives with Single-Walled Carbon Nanotubes for Enhanced Thermoelectric Performance
Source: Molecules. 2023 Sep 8;28(18):6519. doi: 10.3390/molecules28186519 (PMC10534622; doi:10.3390/molecules28186519)
Supplement: Supplementary file 1 [file molecules-28-06519-s001.zip › molecules-2585609-supplementary.pdf]

Supplementary Materials

Compositing Benzothieno[3,2-*b*]Benzofuran Derivatives with Single-Walled Carbon Nanotubes for Enhanced Thermoelectric Performance

Yiyang Li †, Liankun Ai †, Qunyi Luo, Xin Wu, Baolin Li * and Cun-Yue Guo *

School of Chemical Sciences, University of Chinese Academy of Sciences, Beijing 100049, China; liyiyang211@mails.ucas.ac.cn (Y.L.); ailiankun19@mails.ucas.ac.cn (L.A.); luoqunyi20@mails.ucas.ac.cn (Q.L.); wuxin191@mails.ucas.ac.cn (X.W.)

* Correspondence: libl@ucas.ac.cn (B.L.); cyguo@ucas.ac.cn (C.-Y.G.)

† These authors contributed equally to this work.

General Information

Chemicals were purchased from commercial suppliers such as Alfa Aesar, Aladdin, Heowns, Innochem, Meryer or Bidepharm, and used without further purification unless otherwise noted. The synthesis of 2-bromobenzo[4,5]thieno[3,2-*b*]benzofuran had been previously reported by our research group, and the synthesis conditions could be found in the references [S1].

Anhydrous toluene was obtained by distillation over CaH_2. Anhydrous dimethyl sulfoxide was refluxed for 4 h over CaH_2, and then fractionally distilled at low pressure. Thin-layer chromatography was done using TLC silica gel GF254 glass plates and was visualized with a UV lamp at 254 nm and 365 nm. Silica gel (200-300 mesh) was used for flash column chromatography. Proton and carbon nuclear magnetic resonance (^{1}H NMR and ^{13}C NMR) spectra were measured on JEOL 400YH spectrometer at around 18 °C. Chemical shifts (δ) are reported in parts per million (ppm) downfield of tetramethylsilane (TMS, δ = 0), and the residual solvent peaks were used as internal references, for ^{1}H NMR: $CDCl_3$ = 7.26 ppm; for ^{13}C NMR: $CDCl_3$ = 77.16 ppm. The order of citation in parentheses is: a) multiplicity (s = singlet, d = doublet, t = triplet, q = quartet, dd = doublet of doublets, td = triplet of doublets, dt = doublet of triplets, ddd = doublet of doublet of doublets, m = multiplet), b) coupling constants, c) number of protons. Coupling constants (*J*) are reported in Hertz (Hz). The high-resolution mass spectra (HRMS) were conducted on Thermoscientific Q Exactive Focus (ESI).

1. Synthesis details

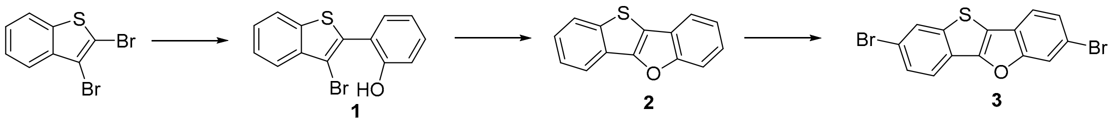

2-(3-bromobenzo[*b*]thiophen-2-yl)phenol (1): 2,3-dibromobenzo[*b*]thiophene (2.92 g, 10.0 mmol), (2-hydroxyphenyl)boronic acid (1.66 g, 12.0 mmol), K_2CO_3 (2.76 g, 20.0 mmol), 1,4-dioxane (75 mL) and water (25 mL) were added to a 250 mL round-bottom flask and purged with N_2. 20 min later, $Pd(PPh_3)_4$ (577.8 mg, 0.50 mmol) was added. The mixture was purged with N_2 for another 15 min and then heated to 90 °C for 12 h. The reaction mixture was cooled, 1 M HCl (20 mL) was added to acidify the solution. Saturated NaCl solution (30 mL) was added and extracted with ethyl acetate (3 × 20 mL). The organic layer was dried over $MgSO_4$, filtered and evaporated. The remaining raw product was purified by silica gel chromatography using petroleum ether (PE)/ethyl acetate (EA) (*v*:*v* = 10:1) as the eluent to give a white powder (2.29 g, 7.50 mmol, yield = 75%). ^{1}H NMR (400 MHz, $CDCl_3$) δ (ppm): 7.92 (d, *J* = 8.1 Hz, 1H), 7.52 (d, *J* = 8.0 Hz, 1H), 7.42 (t, *J* = 7.5 Hz, 1H), 7.34 (t, *J* = 7.5 Hz, 1H), 7.12 (td, *J* = 7.8, 1.9 Hz, 1H), 6.69–6.60 (m, 3H), 4.84 (s, 1H). ^{13}C NMR (101 MHz, $CDCl_3$) δ (ppm): 153.22, 139.81, 139.66, 137.69, 131.32, 130.27, 128.12, 125.16, 124.99, 123.60, 122.66, 120.76, 120.15, 116.18. HRMS (ESI) calculated mass for $C_{14}H_8BrOS$ [M-H]$^-$: 302.9474, found: 302.9474.

Benzo[4,5]thieno[3,2-*b*]benzofuran (2, BTBF) [S2,S3]: 2-(3-bromobenzo[*b*]thiophen-2-yl)phenol (1.22 g, 4.0 mmol), K_2CO_3 (1.10 g, 8.0 mmol), CuI (152.4 mg, 0.8 mmol), 1,10-phenanthroline (144.2 mg, 0.8 mmol) and DMF (20 mL) were added to a 50 mL Schlenk flask and purged with N_2. The mixture was purged with N_2 for 15 min and then heated to 90 °C. Three hours later, the reaction mixture was cooled and 1 M HCl (10 mL) was added to acidify the solution. Saturated NaCl solution (30 mL) was added and extracted with ethyl acetate (3 × 15 mL). The organic layer was dried over $MgSO_4$, filtered and evaporated. The remaining crude product was purified by silica gel chromatography using PE as eluent to give a white powder (663.0 mg, 2.96 mmol, yield = 74%). ^1H NMR (400 MHz, $CDCl_3$) δ (ppm): 8.02 (d, J = 8.1 Hz, 1H), 7.89 (d, J = 8.1 Hz, 1H), 7.74 (d, J = 8.0 Hz, 1H), 7.66 (d, J = 8.0 Hz, 1H), 7.53–7.46 (m, 1H), 7.42–7.32 (m, 3H). ^{13}C NMR (101 MHz, $CDCl_3$) δ (ppm): 158.90, 153.12, 142.11, 125.25, 125.07, 125.04 (one carbon was overlapped), 124.49, 124.17, 123.43, 119.82, 119.72, 118.70, 112.69. HRMS (ESI) calculated mass for $C_{14}H_8OS$ $[M]^+$: 224.0290, found 224.0289.

2,7-dibromobenzo[4,5]thieno[3,2-*b*]benzofuran (3, BTBF-2Br): benzo[4,5]thieno[3,2-*b*]benzofuran (448 mg, 2 mmol), potassium acetate (490.7 mg, 5 mmol), trichloromethane (30 mL) were added to a 100 mL round-bottom flask, and then reaction mixture was cooled with ice water bath. Br_2 (0.26 mL, 5 mmol) was diluted with trichloromethane (5 mL) and added dropwise. The reaction system was heated to 70 °C for 24 h. After being allowed to cool to room temperature, added saturated $NaHSO_3$ solution (20 mL) to quench Br_2. After the addition of saturated NaCl solution (10 mL) and extraction with dichloromethane (DCM, 3 × 10 mL), the organic layer was dried over $MgSO_4$, filtered and evaporated. The remaining raw product was purified by silica gel chromatography using petroleum ether (PE) as the eluent to give a white powder (489 mg, 1.28 mmol, yield = 64%). ^1H NMR (400 MHz, $CDCl_3$) δ (ppm): 8.01 (d, J = 1.8 Hz, 1H), 7.83 (d, J = 8.6 Hz, 1H), 7.80 (d, J = 1.7 Hz, 1H), 7.58 (dd, J = 8.6, 1.8 Hz, 1H), 7.56 (d, J = 8.5 Hz, 1H) 7.47 (dd, J = 8.5, 1.7 Hz, 1H). ^{13}C NMR (101 MHz, $CDCl_3$) δ (ppm): 159.20, 152.98, 143.58, 128.78, 127.13, 127.10, 123.84, 123.07, 120.87, 120.56, 119.07, 118.92, 118.56, 116.31. HRMS (ESI) calculated mass for $C_{14}H_6Br_2SO$ $[M]^+$: 379.8501, found: 379.8488.

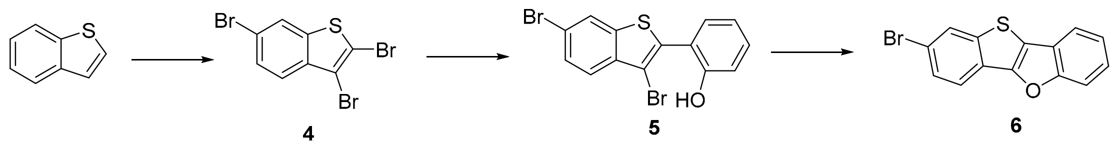

2,3,6-tribromobenzo[*b*]thiophene (4): Benzo[*b*]thiophene (5.01 g, 37.3 mmol), potassium acetate (16.45 g, 167.9 mmol), trichloromethane (100 mL) were added to a 250 mL round-bottom flask, and then reaction mixture was cooled with ice water bath. Br_2 (8.60 mL, 167.9 mmol) was diluted with trichloromethane (20 mL) and added dropwise. The reaction system was heated to 70 °C for 24 h. After being allowed to cool to room temperature, added saturated $NaHSO_3$ solution (20 mL) to quench Br_2.

After the addition of saturated NaCl solution (30 mL) and extraction with dichloromethane (DCM, 3 × 30 mL), the organic layer was dried over $MgSO_4$, filtered and evaporated. The remaining raw product was purified by silica gel chromatography using petroleum ether (PE) as the eluent to give a white powder (11.62 g, 31.6 mmol, yield = 85%). ^{1}H NMR (400 MHz, $CDCl_3$) δ (ppm): 7.87 (d, J = 1.7 Hz, 1H), 7.60 (d, J = 8.6 Hz, 1H), 7.54 (dd, J = 8.6, 1.7 Hz, 1H). ^{13}C NMR (101 MHz, $CDCl_3$) δ (ppm): 140.15, 136.49, 129.19, 124.55, 124.48, 119.92, 114.88, 111.71. HRMS (ESI) calculated mass for $C_8H_3Br_3S$ $[M]^+$: 367.7500, found: 367.7495.

2-(3,6-dibromobenzo[*b*]thiophen-2-yl)phenol (5): 2,3,6-tribromobenzo[*b*]thiophene (3.71 g, 10.0 mmol), (2-hydroxyphenyl)boronic acid (1.66 g, 12.0 mmol), K_2CO_3 (2.76 g, 20.0 mmol), 1,4-dioxane (75 mL) and water (25 mL) were added to a 250 mL round-bottom flask and purged with N_2. 20 min later, $Pd(PPh_3)_4$ (577.8 mg, 0.50 mmol) was added. The mixture was purged with N_2 for another 15 min and then heated to 90 °C for 12 h. The reaction mixture was cooled, 1 M HCl (20 mL) was added to acidify the solution. Saturated NaCl solution (30 mL) was added and extracted with ethyl acetate (3 × 20 mL). The organic layer was dried over $MgSO_4$, filtered and evaporated. The remaining raw product was purified by silica gel chromatography using petroleum ether (PE)/ethyl acetate (EA) (v:v = 10:1) as the eluent to give a white powder (2.88 g, 7.50 mmol, yield = 75%). ^{1}H NMR (400 MHz, $CDCl_3$) δ (ppm): 7.99 (d, J = 1.6 Hz, 1H), 7.73 (d, J = 8.6 Hz, 1H), 7.61 (dd, J = 8.7, 1.9 Hz, 1H), 7.45–7.32 (m, 2H), 7.14–6.99 (m, 2H), 5.18 (s, 1H). ^{13}C NMR (101 MHz, $CDCl_3$) δ (ppm): 153.36, 140.17, 137.36, 134.30, 131.81, 131.59, 129.10, 125.01, 124.96, 120.97, 120.08, 118.70, 116.58, 108.39. HRMS (ESI) calculated mass for $C_{14}H_7Br_2OS$ $[M-H]^-$: 380.8579, found: 380.8601.

2-bromobenzo[4,5]thieno[3,2-*b*]benzofuran (6, BTBF-Br): 2-(3,6-dibromobenzo[*b*]thiophen-2-yl)phenol (1.54 g, 4.0 mmol), K_2CO_3 (1.10 g, 8.0 mmol), CuI (152.4 mg, 0.8 mmol), 1,10-phenanthroline (144.2 mg, 0.8 mmol) and DMF (20 mL) were added to a 50 mL Schlenk flask and purged with N_2. The mixture was purged with N_2 for 15 min and then heated to 90 °C. Three hours later, the reaction mixture was cooled, 1 M HCl (10 mL) was added to acidify the solution. Saturated NaCl solution (30 mL) was added and extracted with ethyl acetate (3 × 15 mL). The organic layer was dried over $MgSO_4$, filtered and evaporated. The remaining crude product was purified by silica gel chromatography using PE as eluent to give a white powder (892.4 mg, 2.96 mmol, yield = 74%). ^{1}H NMR (400 MHz, $CDCl_3$) δ (ppm): 8.00 (d, J = 1.6 Hz, 1H), 7.83 (d, J = 8.4 Hz, 1H), 7.71 (dd, J = 8.0, 1.6Hz, 1H), 7.63 (dd, J = 8.0, 1.2Hz, 1H), 7.57 (dd, J = 8.4, 1.2 Hz, 1H), 7.40−7.35 (m, 2H). ^{13}C NMR (101 MHz, $CDCl_3$) δ (ppm): 159.01, 152.48, 143.41, 128.54, 127.03, 125.46, 124.05, 123.93, 123.67, 120.78, 119.82, 119.23, 118.59, 112.80. HRMS (ESI) calculated mass for $C_{14}H_7BrOS$ $[M]^+$: 301.9395, found: 301.9391.

2. NMR spectra

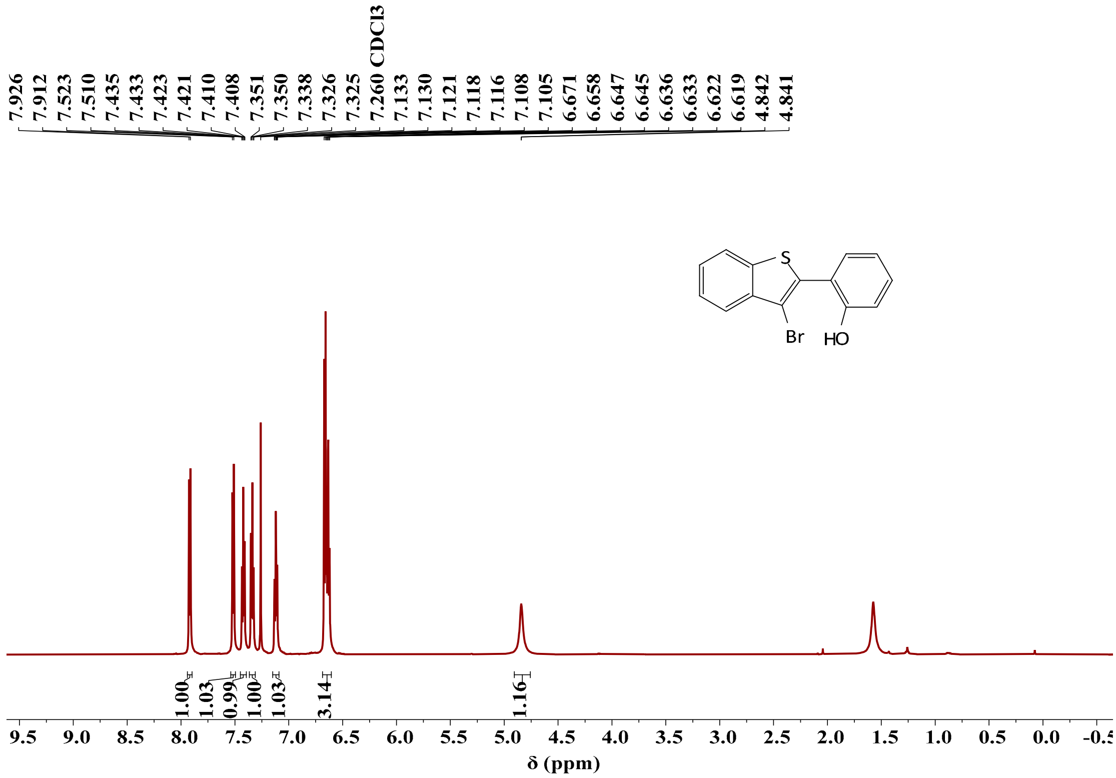

Figure S1. ^{1}H NMR spectrum of **1**.

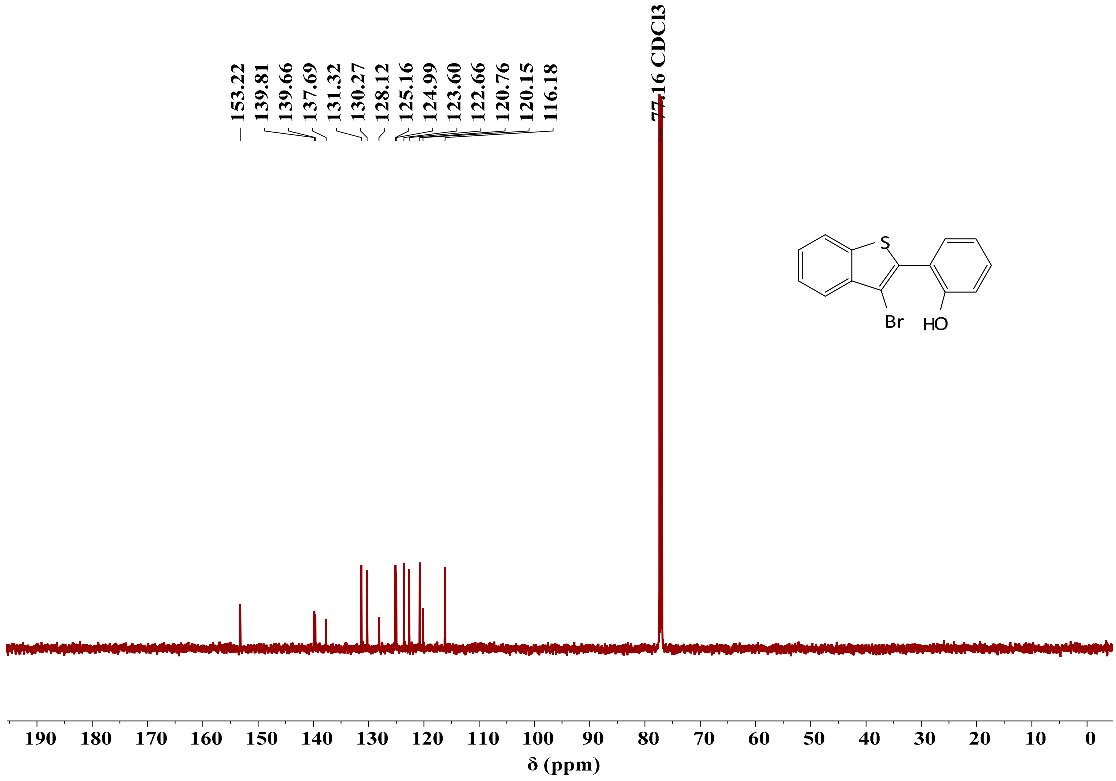

Figure S2. ^{13}C NMR spectrum of **1**.

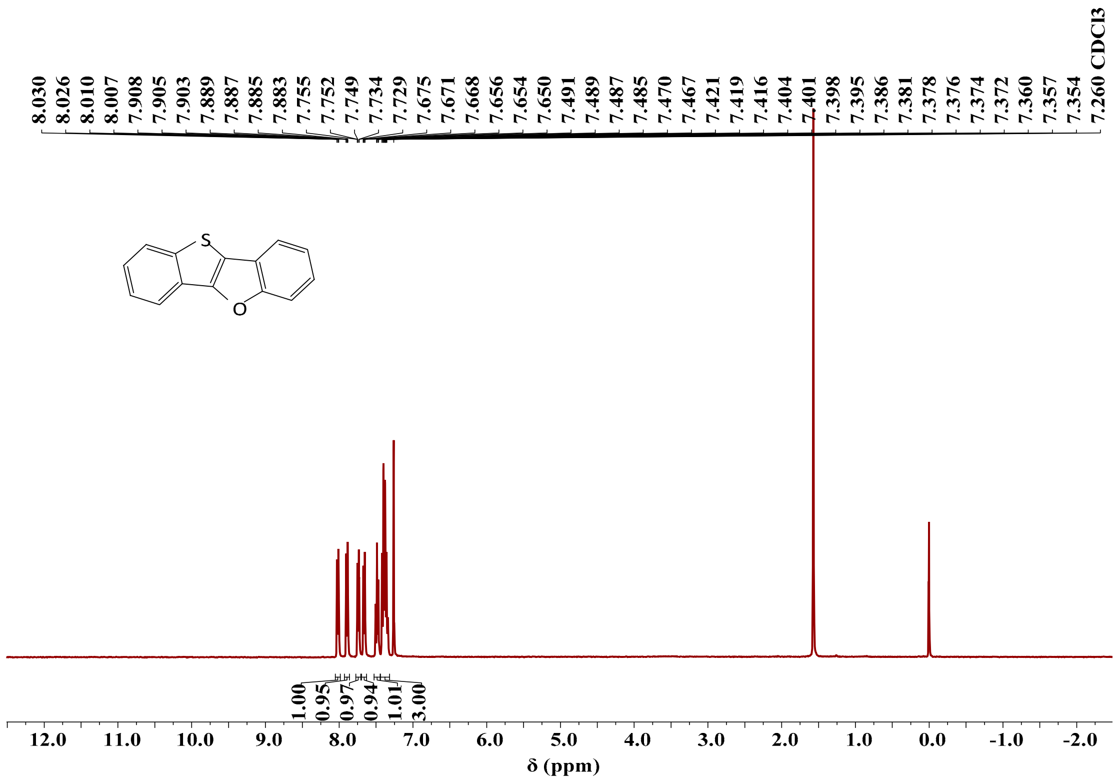

Figure S3. ^{1}H NMR spectrum of **2**.

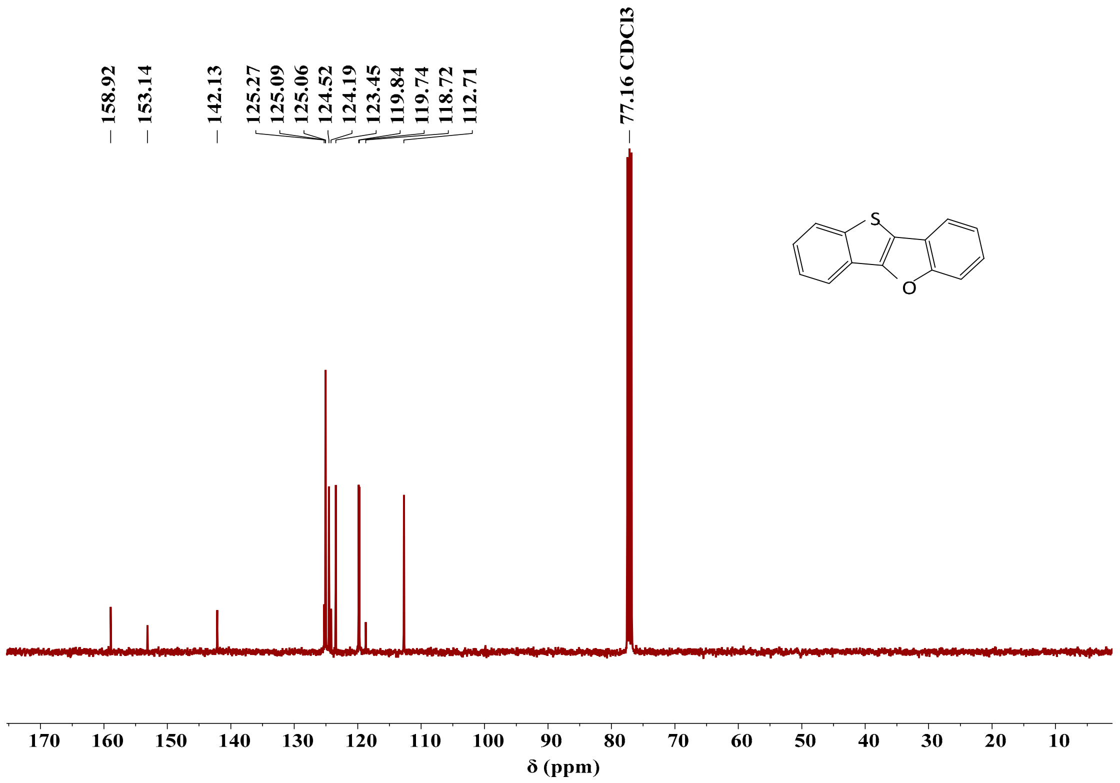

Figure S4. ^{13}C NMR spectrum of **2**.

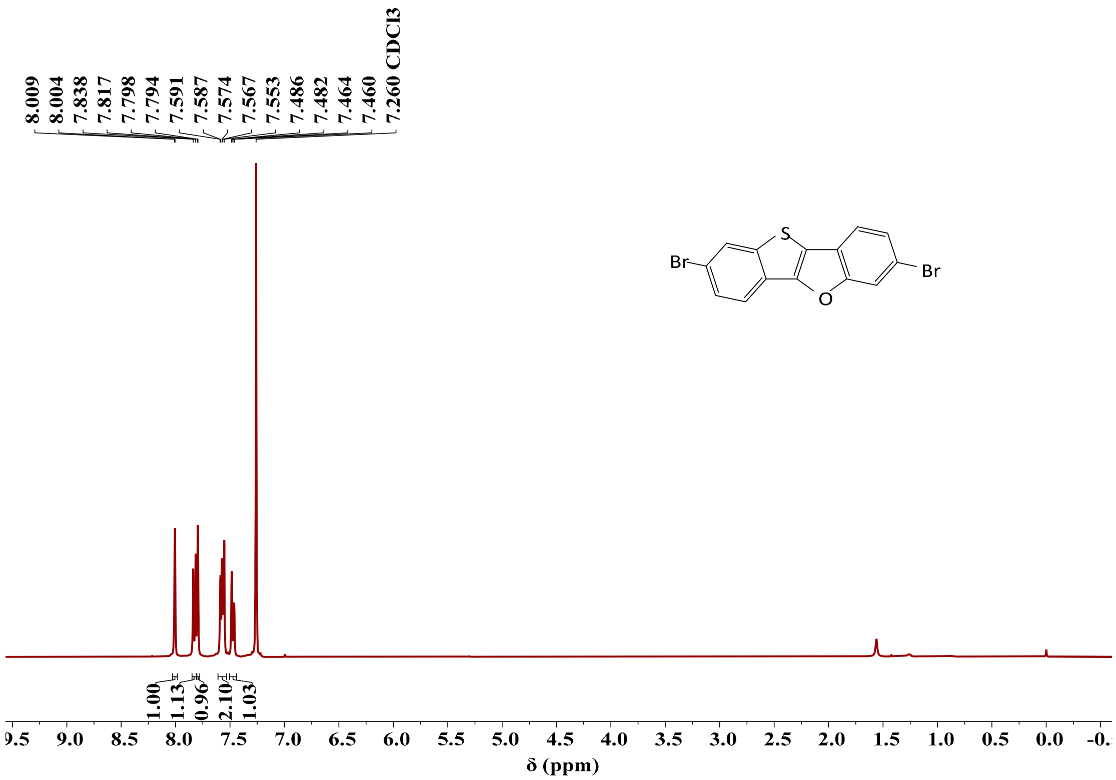

Figure S5. ^{1}H NMR spectrum of **3**.

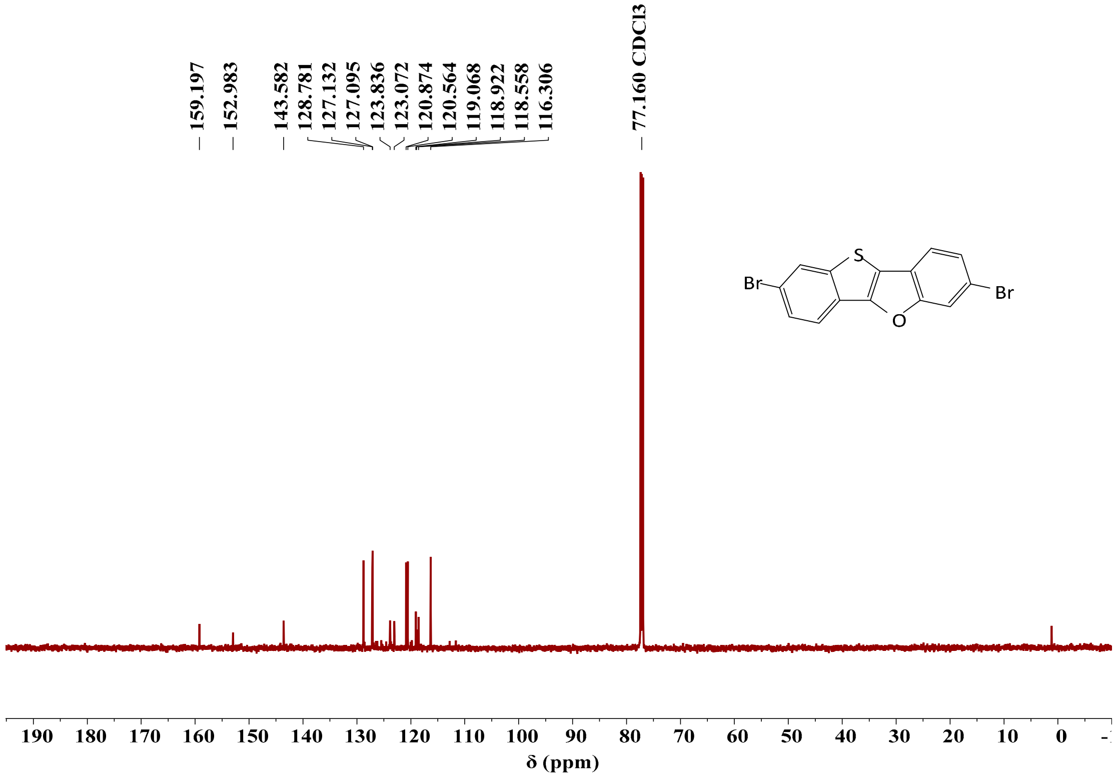

Figure S6. ^{13}C NMR spectrum of **3**.

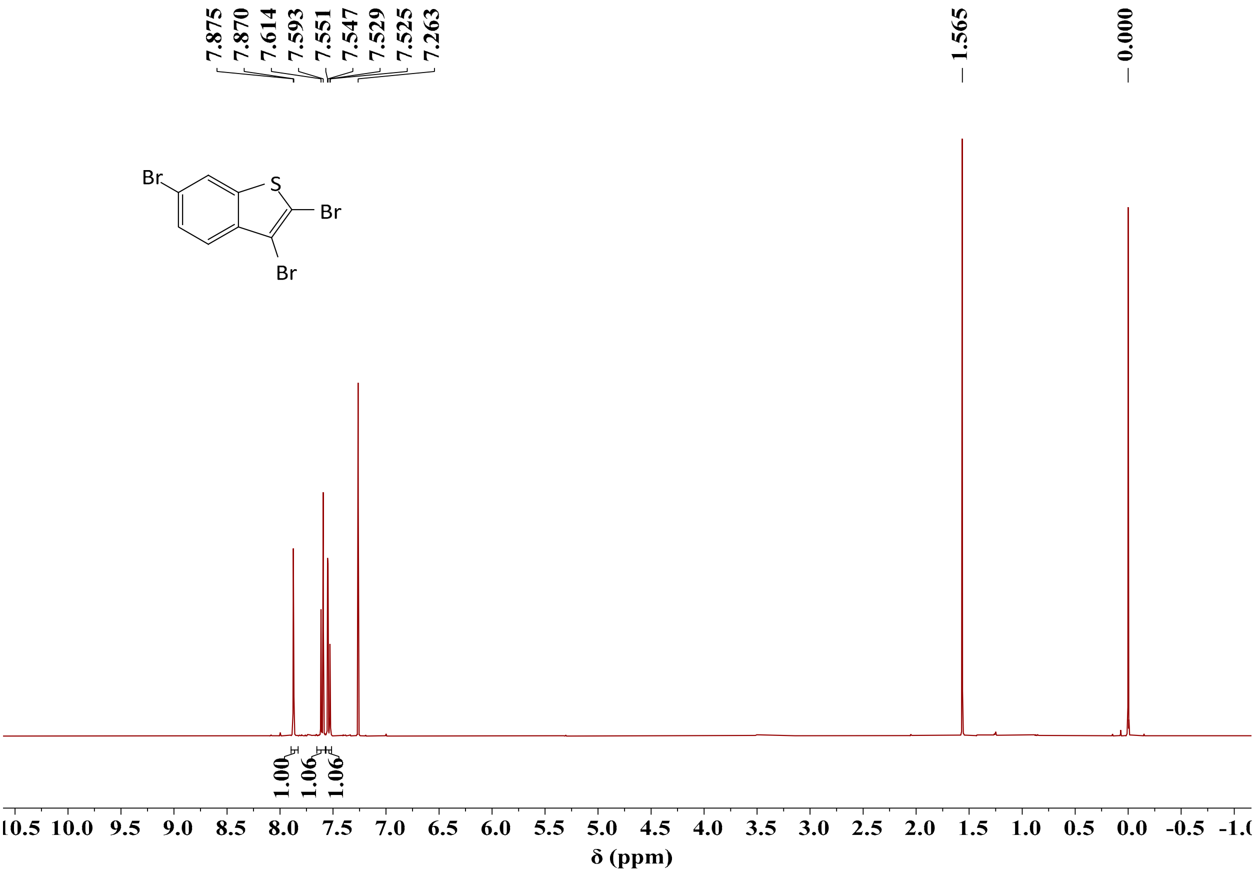

Figure S7. ^{1}H NMR spectrum of **4**.

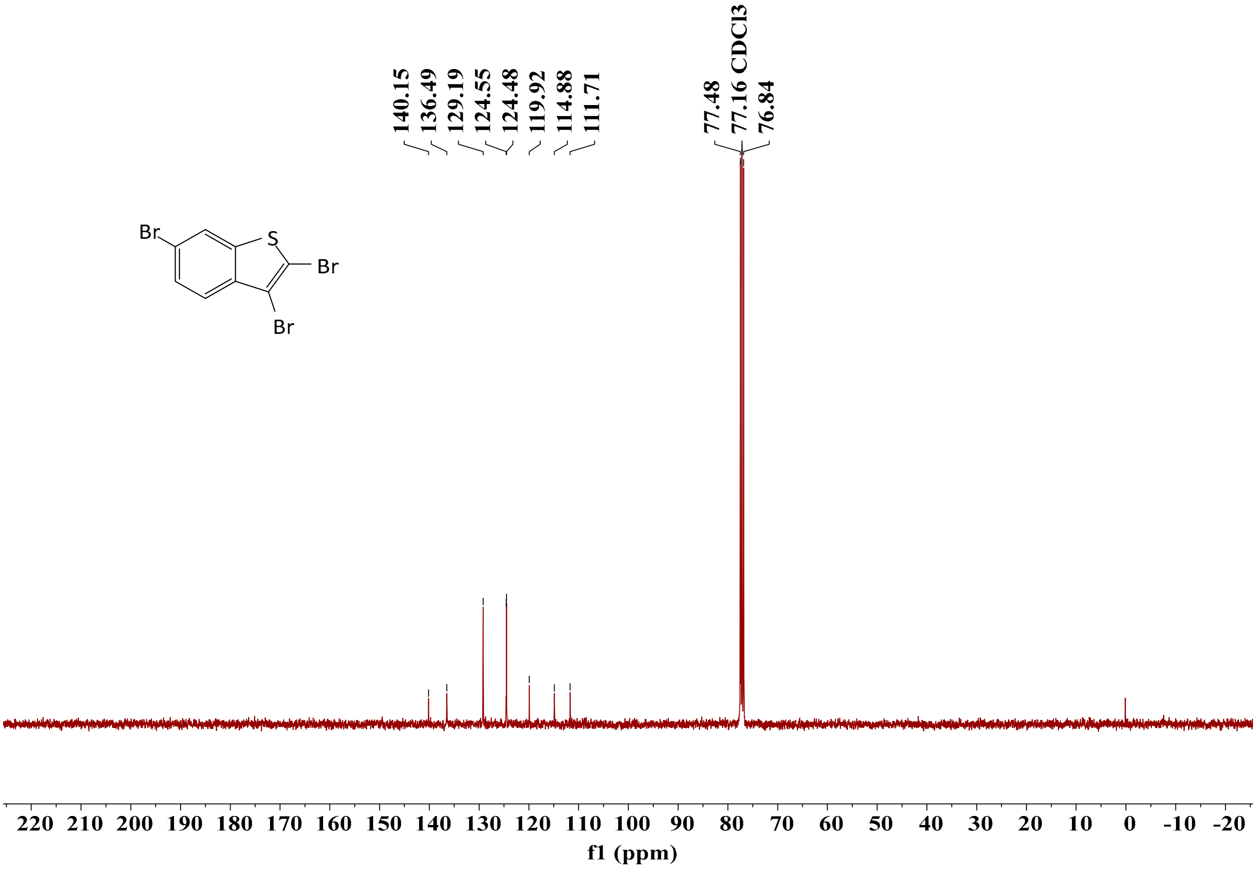

Figure S8. ^{13}C NMR spectrum of **4**.

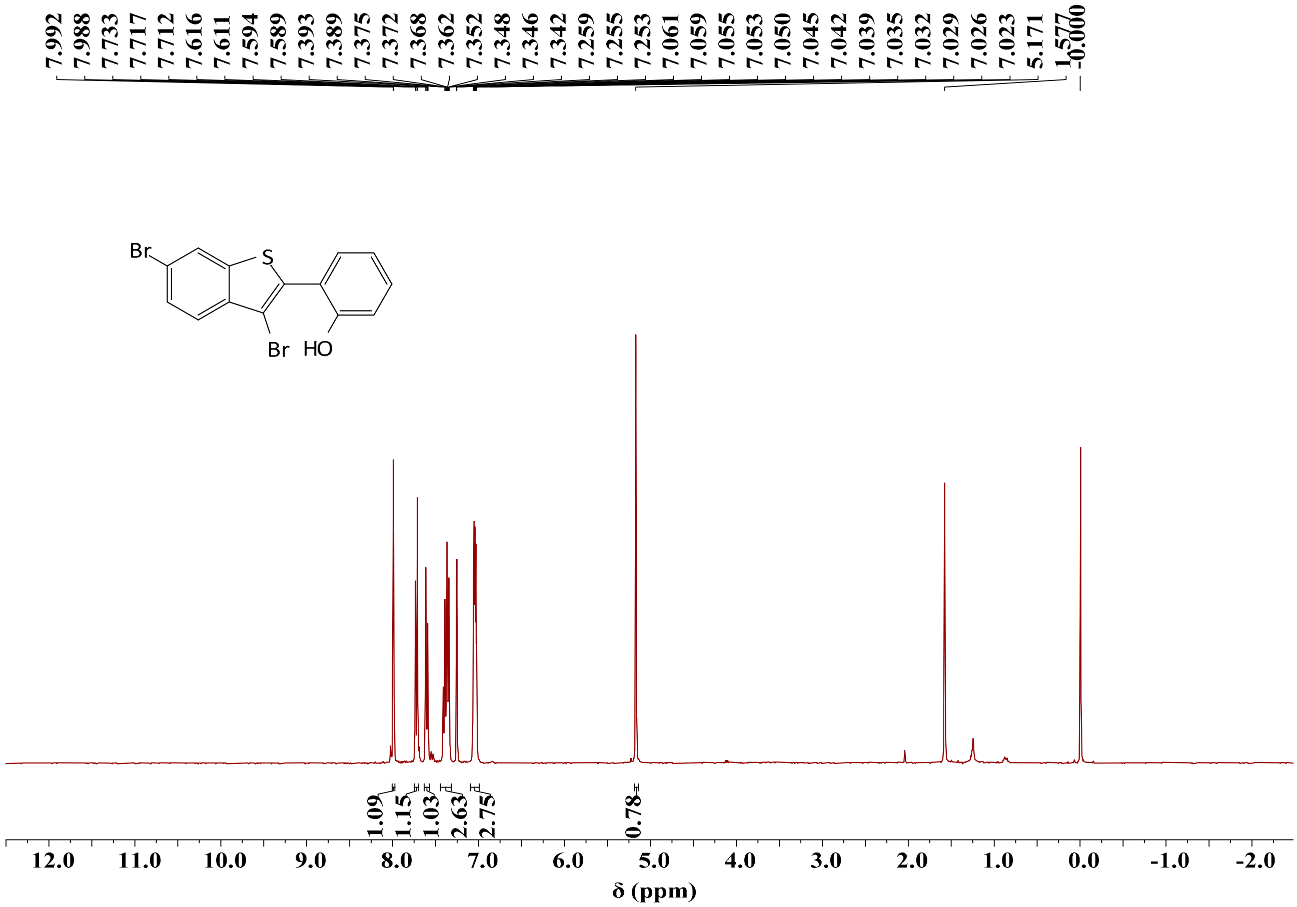

Figure S9. ^{1}H NMR spectrum of **5**.

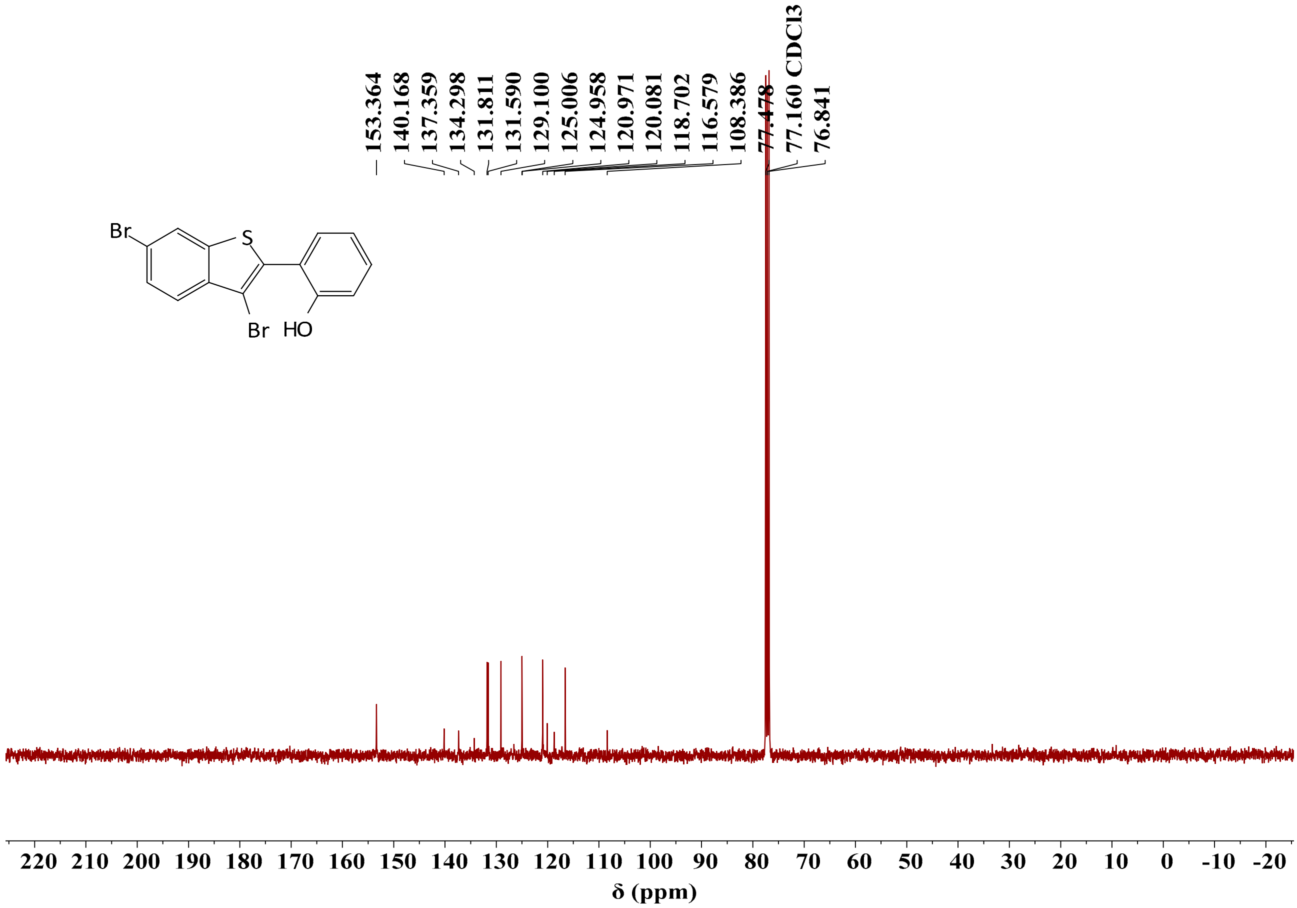

Figure S10. ^{13}C NMR spectrum of **5**.

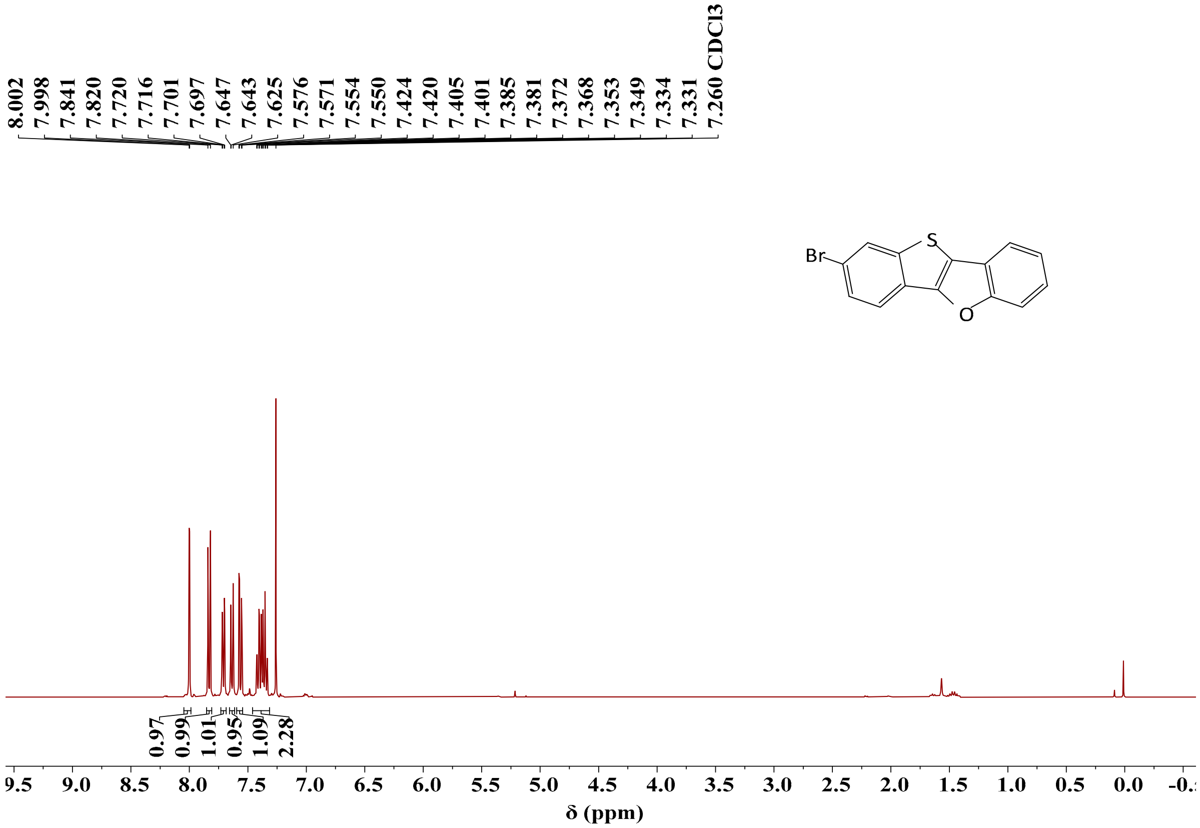

Figure S11. ^{1}H NMR spectrum of **6**.

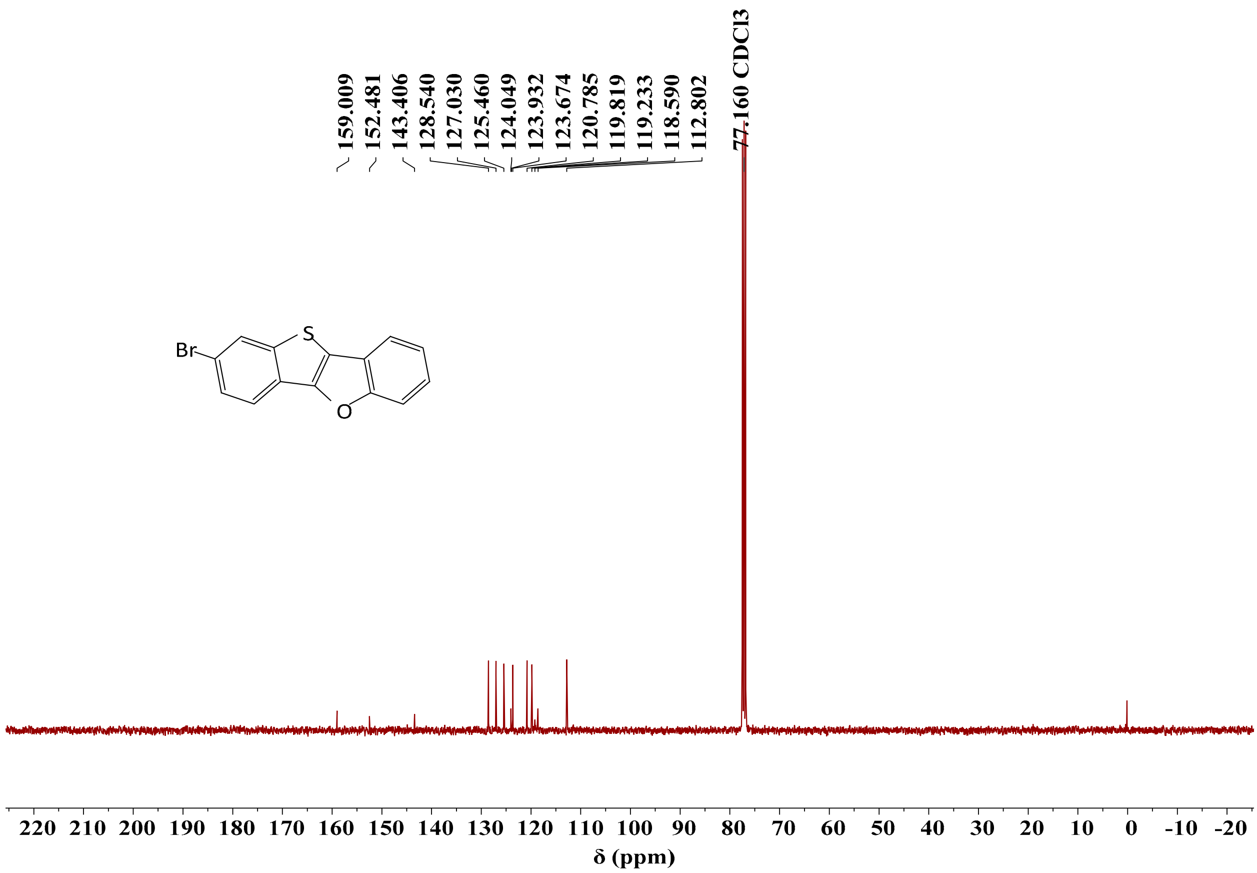

Figure S12. ^{13}C NMR spectrum of **6**.

3. HRMS spectra

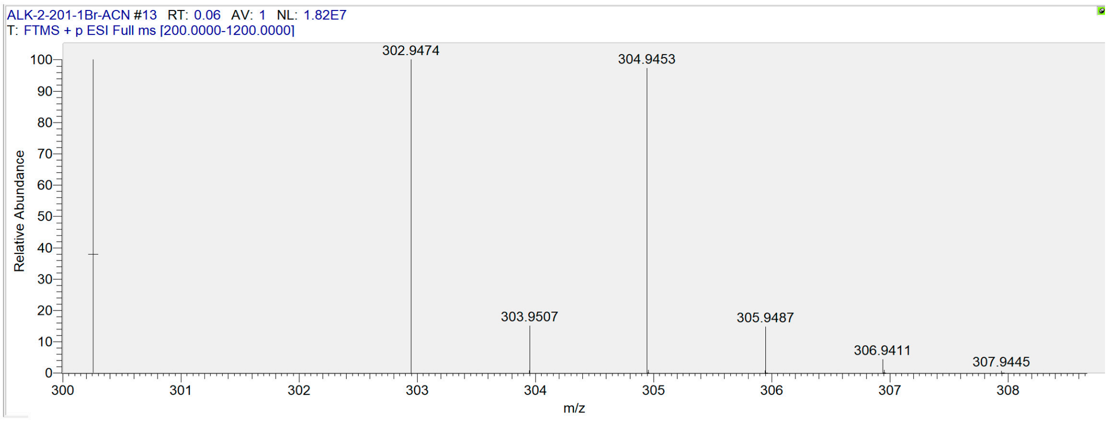

Figure S13. HRMS spectrum of **1**.

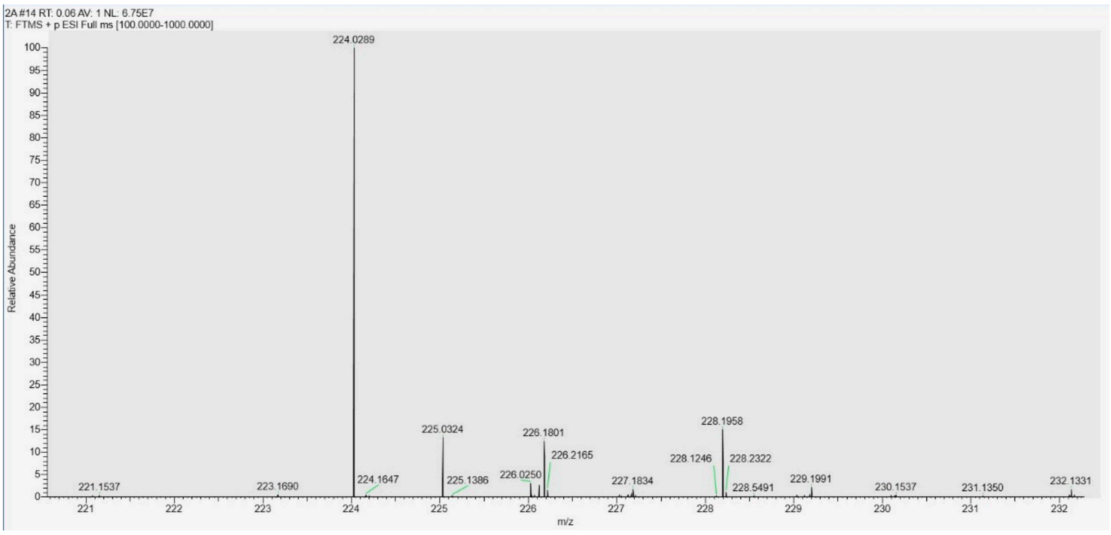

Figure S14. HRMS spectrum of **2**.

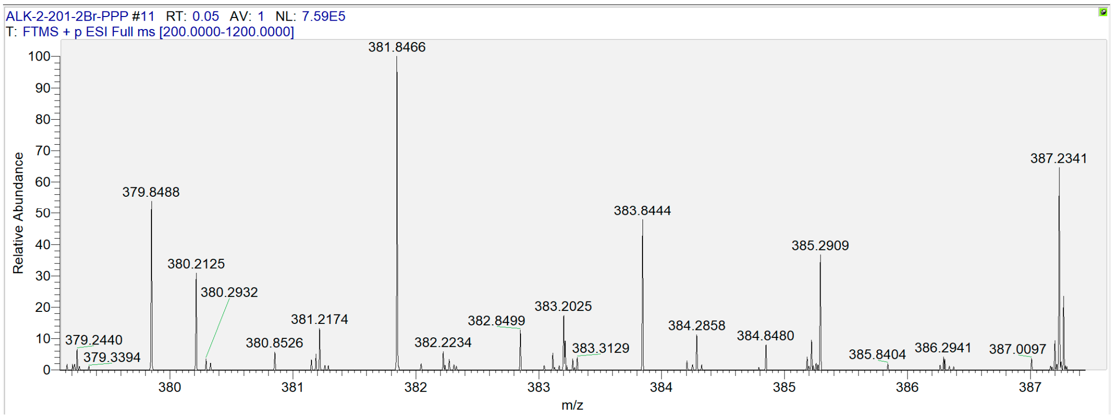

Figure S15. HRMS spectrum of **3**.

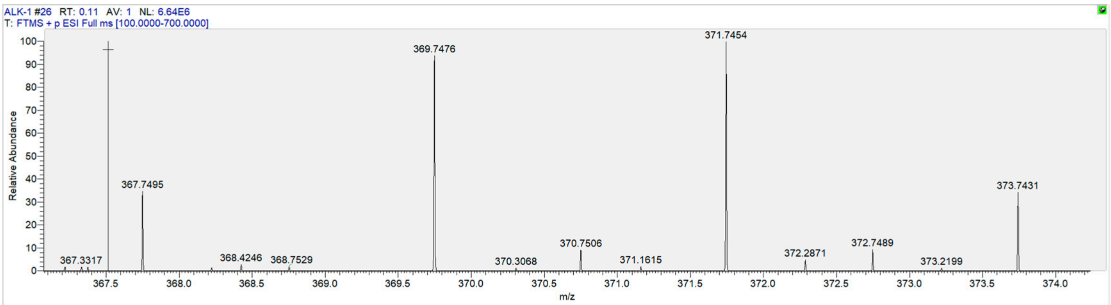

Figure S16. HRMS spectrum of **4**.

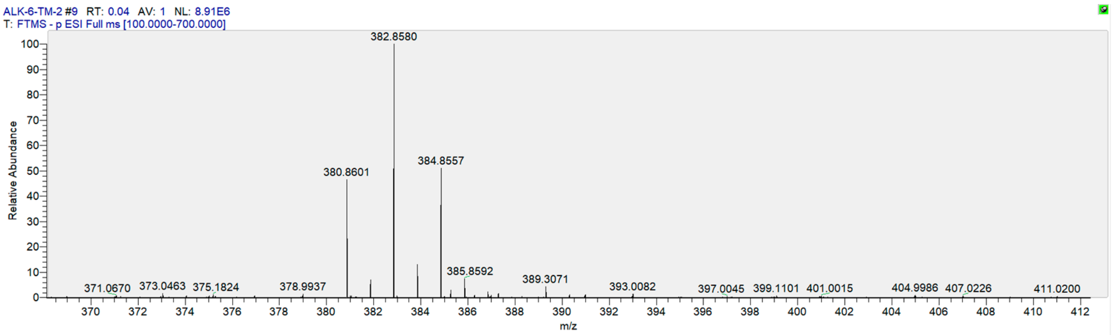

Figure S17. HRMS spectrum of **5**.

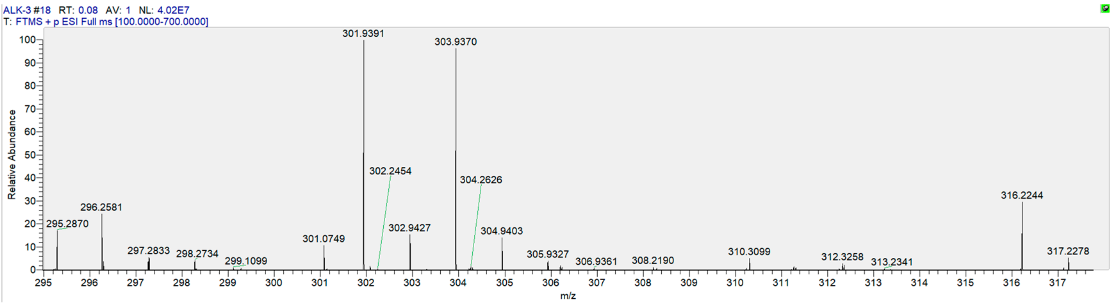

Figure S18. HRMS spectrum of **6**.

4. FT-IR spectra of OSM/SWCNT composite films

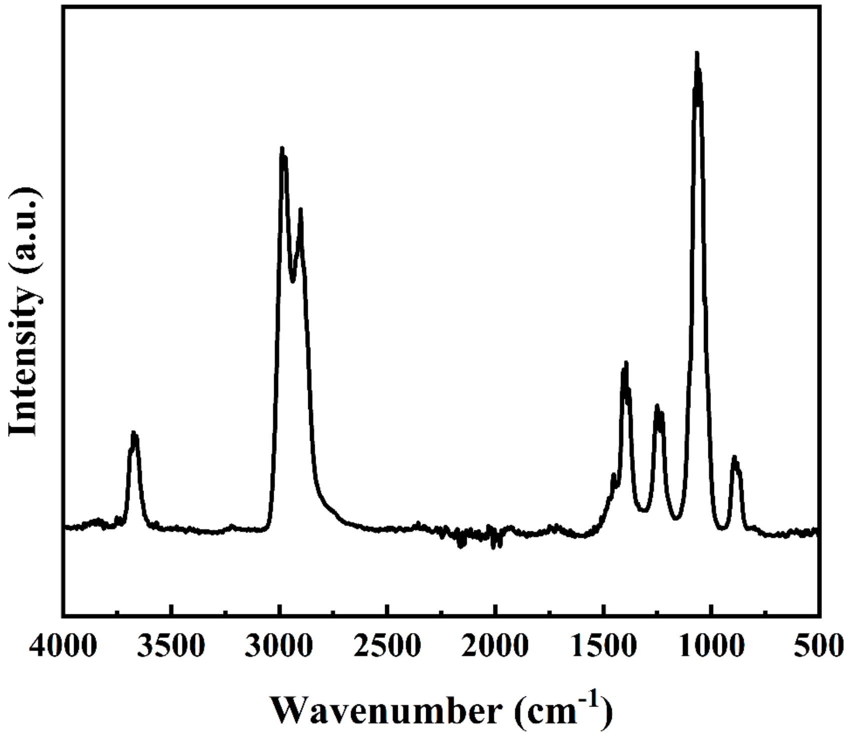

Figure S19. FT-IR spectra of BTBF/SWCNT composite film.

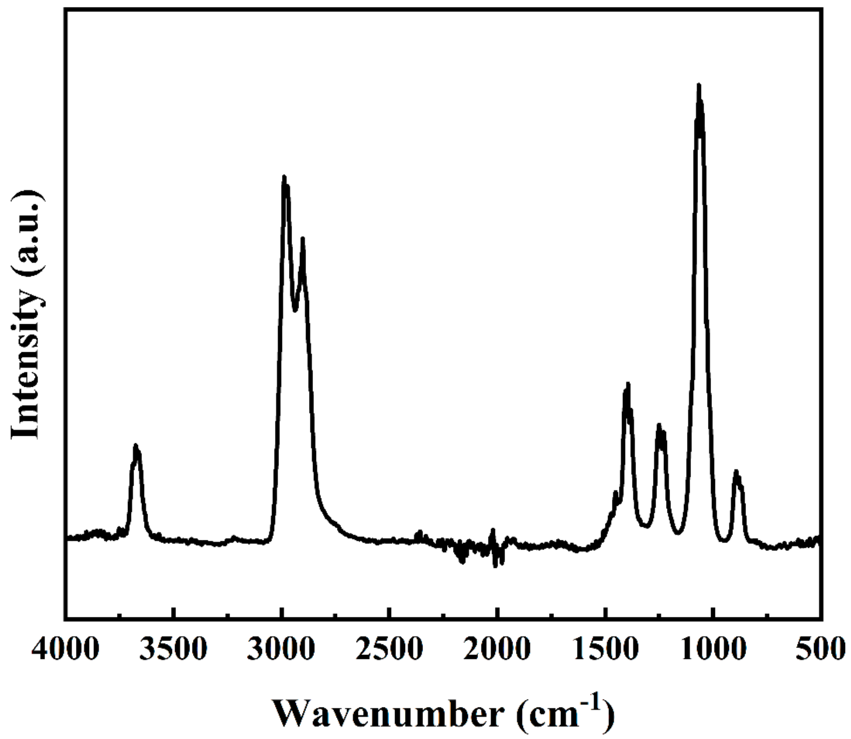

Figure S20. FT-IR spectra of BTBF-Br/SWCNT composite film.

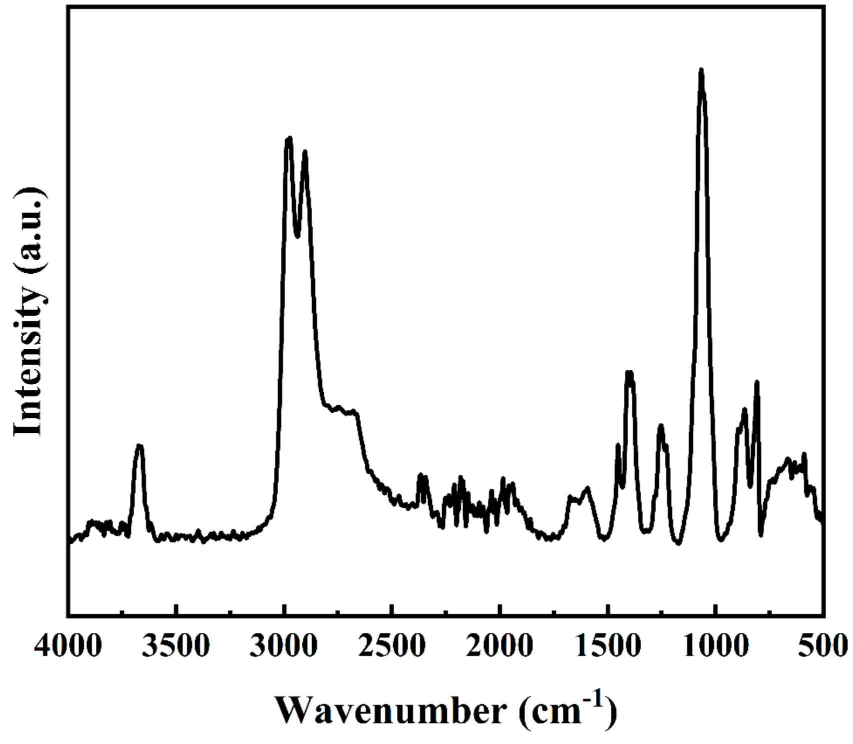

Figure S21. FT-IR spectra of BTBF-2Br/SWCNT composite film.

5. SEM images and EDS images of OSM/SWCNT composite films

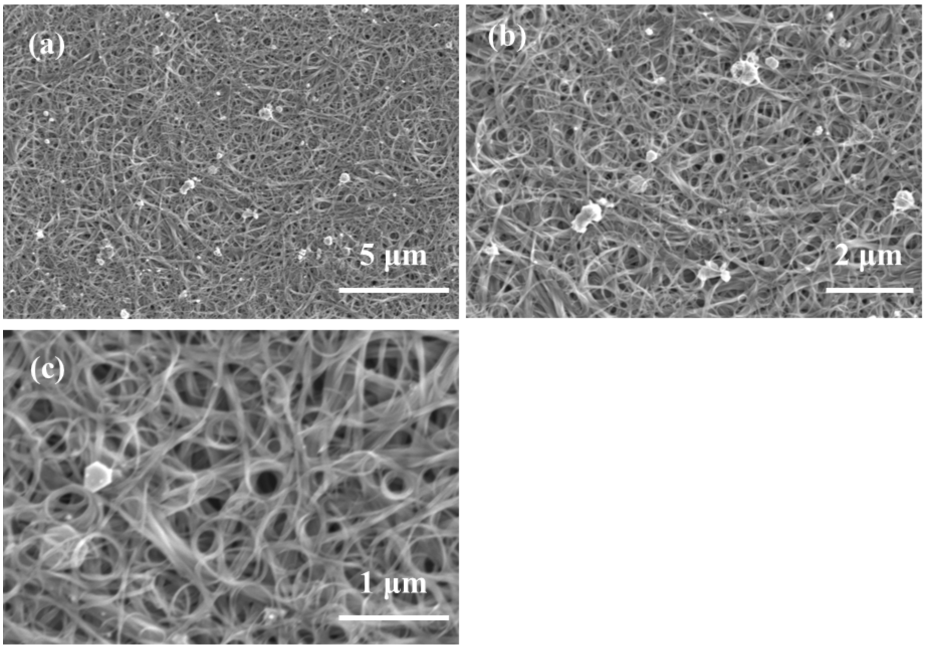

Figure S22. SEM images of 50 wt% BTBF/SWCNT composite film at 6k (a), 12k (b), and 30k (c) magnification.

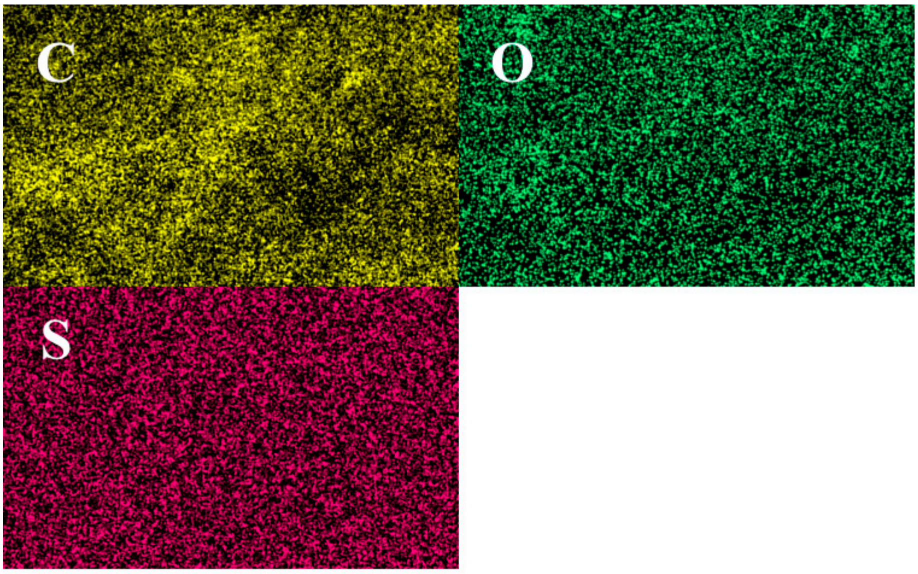

Figure S23. Elemental mapping patterns of 50 wt% BTBF/SWCNT at 12k magnification.

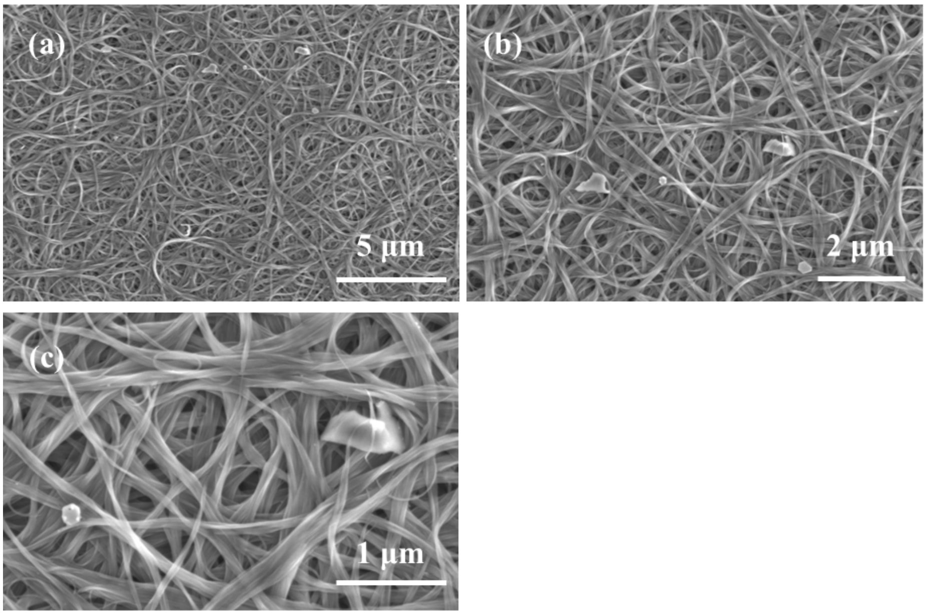

Figure S24. SEM images of 50 wt% BTBF-Br/SWCNT composite film at 6k (a), 12k (b), and 30k (c) magnification.

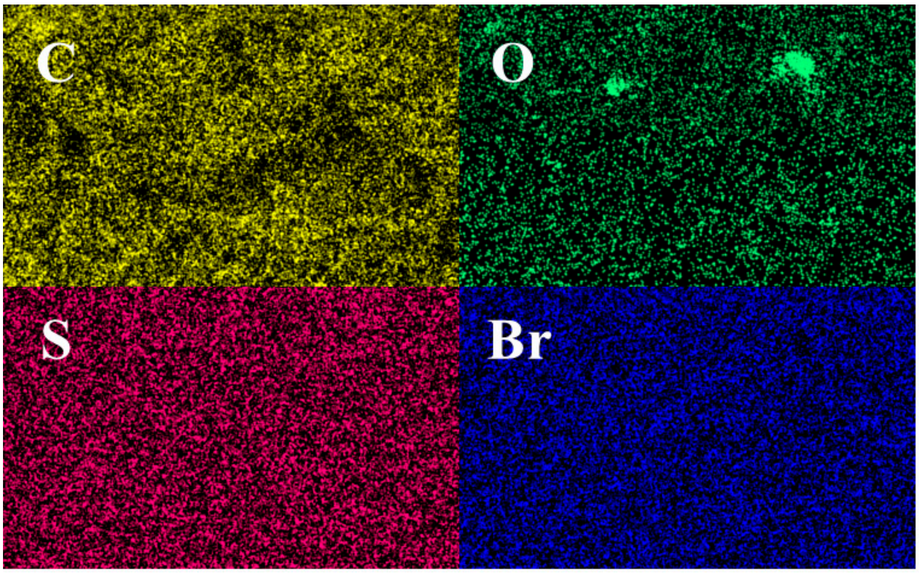

Figure S25. Elemental mapping patterns of 50 wt% BTBF-Br/SWCNT at 12k magnification.

6. Raman Spectroscopy of OSM/SWCNT composite films

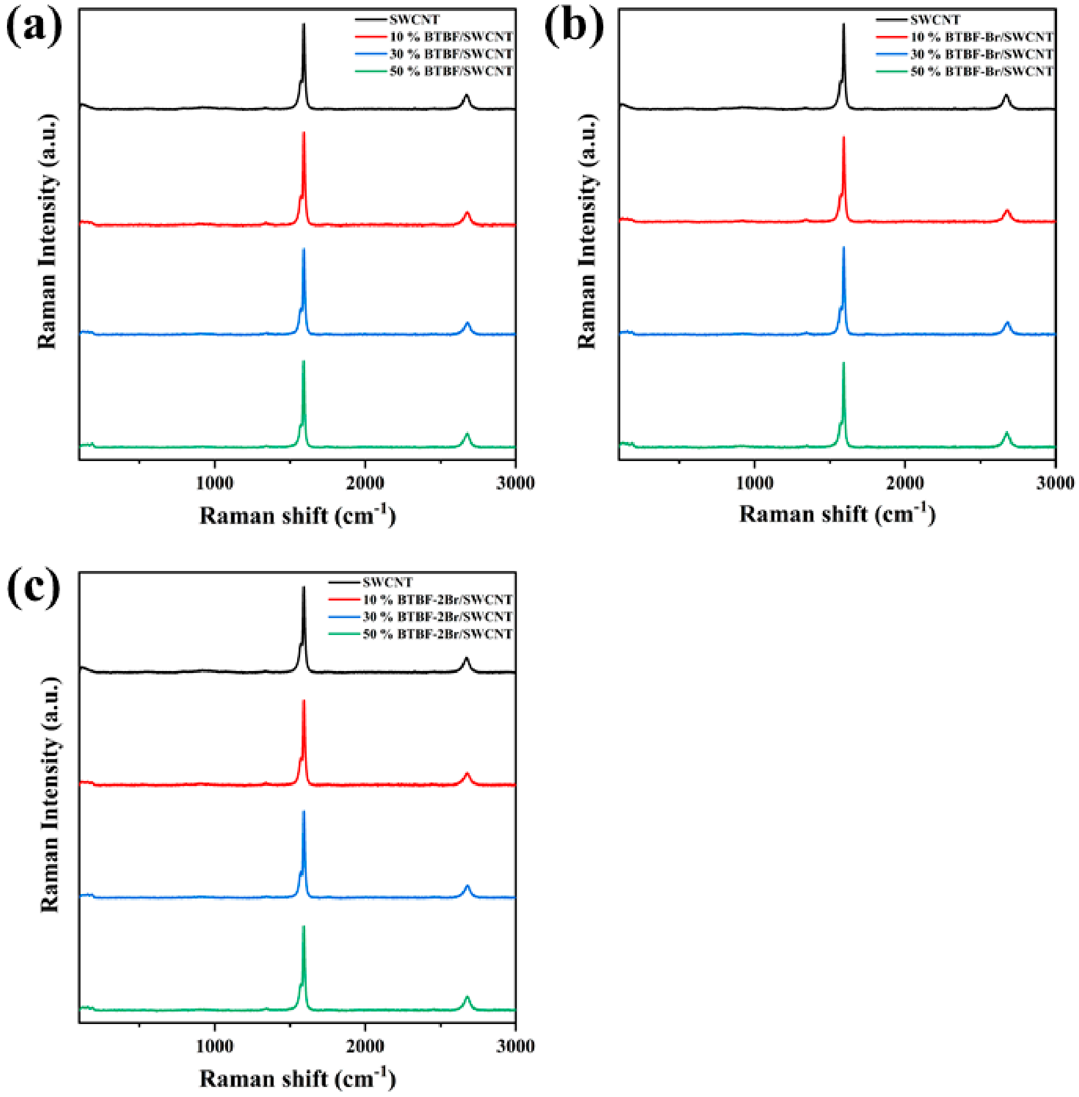

Figure S26. Raman spectra of pure SWCNT, BTBF/SWCNT (a), BTBF-Br/SWCNT (b), and BTBF-2Br/SWCNT (c).

Table S1. Comparison of thermoelectric properties of representative OSM/SWCNT composites at room temperature

Composites	S (μV K^{-1})	σ (S cm^{-1})	PF (μW m^{-1} K^{-2})	Ref.
BTBF-2Br/SWCNT	56.5 ± 0.5	530.8 ± 18.7	169.7 ± 3.4	This work
HAT6/SWCNT	56.4 ± 4.7	1286.8 ± 152.3	408.2 ± 48.9	[S4]
TPNO/SWCNT	27.7 ± 0.5	382.8 ± 8.9	29.4 ± 1.0	[S5]
Por-5F/SWCNT	53.3 ± 0.4	982.4 ± 20.4	279.3 ± 9.8	[S6]
TczPy/SWCNT	75.9 ± 3.3	189.4 ± 9.0	108.4 ± 4.8	[S7]
NDI/p-SWCNT	44.8 ± 0.4	1316.4 ± 2.5	277.1 ± 5.4	[S8]

7. References

[S1] Ai, L., Xie, X., Li, B., Wang, Y., Pure blue emitters based on benzo[4,5]thieno-S,S-dioxide-[3, 2-b]benzofuran with high thermal stability. *Dyes Pigments* **2023,** *213*, 111153.

[S2] Chen, D., Yuan, D., Zhang, C., Wu, H., Zhang, J., Li, B., Zhu, X., Ullmann-type intramolecular C–O reaction toward thieno[3, 2-b]furan derivatives with up to six fused rings. *J. Org. Chem.* **2017,** *82*, 10920-10927.

[S3] Ma, W., Huang, J., Li, C., Jiang, Y., Li, B., Qi, T., Zhu, X., One-pot synthesis and property study on thieno[3, 2-b]furan compounds. *RSC Adv.* **2019,** *9*, 7123-7127.

[S4] Li, X.; Yu, Z.; Zhou, H.; Yang, F.; Zhong, F.; Mao, X.; Li, B.; Xin, H.; Gao, C.; Wang, L., Promoting the thermoelectric performance of single-walled carbon nanotubes by inserting discotic liquid-crystal molecules. *ACS Sustainable Chem. Eng.* **2021,** *9*, 1891-1898.

[S5] Gao, C.; Chen, G., In situ oxidation synthesis of p-type composite with narrow-bandgap small organic molecule coating on sin-gle-walled carbon nanotube: Flexible film and thermoelectric performance. *Small* **2018**, *14*, e1703453.

[S6] Zhou, Y.; Yin, X.; Liu, Y.; Zhou, X.; Wan, T.; Wang, S.; Gao, C.; Wang, L., Significantly enhanced power factors of p-type carbon nanotube-based composite films by tailoring the peripheral substituents in porphyrin. *ACS Sustainable Chem. Eng.* **2019**, *7*, 11832-11840.

[S7] Yin, X.; Peng, Y.; Luo, J.; Zhou, X.; Gao, C.; Wang, L.; Yang, C., Tailoring the framework of organic small molecule semiconductors towards high-performance thermoelectric composites via conglutinated carbon nanotube webs. *J. Mater. Chem. A* **2018,** *6*, 8323-8330.

[S8] Wang, Y.; Chen, Z.; Huang, H.; Wang, D.; Liu, D.; Wang, L., Organic radical compound and carbon nanotube composites with enhanced electrical conductivity towards high-performance p-type and n-type thermoelectric materials. *J. Mater. Chem. A* **2020,** *8*, 24675-24684.
